# Targeting FTO induces colorectal cancer ferroptotic cell death by decreasing SLC7A11/GPX4 expression

**DOI:** 10.1186/s13046-024-03032-9

**Published:** 2024-04-10

**Authors:** Yaya Qiao, Meng Su, Huifang Zhao, Huanle Liu, Chenxi Wang, Xintong Dai, Lingling Liu, Guangju Liu, Huanran Sun, Mingming Sun, Jiyan Wang, Zhen Li, Jun Fan, Quan Zhang, Chunshen Li, Fangmin Situ, Jun Xue, Zhenghu Jia, Chunze Zhang, Shuai Zhang, Changliang Shan

**Affiliations:** 1 State Key Laboratory of Medicinal Chemical Biology, College of Pharmacy and Tianjin Key Laboratory of Molecular Drug Research, Nankai University, Tianjin, 300350 China; 2 School of Life Science and Bio-pharmaceutics, Shenyang Pharmaceutical University, Liaoning Shenyang, 117004 China; 3 School of Integrative Medicine, Tianjin University of Traditional Chinese Medicine, Tianjin, 301617 China; 4 Guangzhou key laboratory for clinical rapid diagnosis and early warning of infectious diseases, KingMed School of Laboratory Medicine, Guangzhou Medical University, Guangdong Guangzhou, 510180 China; 5 Department of Medical Biochemistry and Molecular Biology, School of Medicine, Guangdong Second Provincial General Hospital, Jinan University, Guangzhou, 510632 China; 6 College of Chinese and Culture, Jinan University, Guangzhou, 510632 China; 7 Department of General Surgery, The First Affiliated Hospital of Hebei North University, Zhangjiakou, 075000 China; 8 The First Affiliated Hospital, Biomedical Translational Research Institute and Guangdong Province Key Laboratory of Molecular Immunology and Antibody Engineering, Jinan University, Guangzhou, 510632 China; 9 Tianjin Key Laboratory for Modern Drug Delivery & High-Efficiency, Collaborative Innovation Center of Chemical Science and Engineering, School of Pharmaceutical Science and Technology, Tianjin University, Tianjin, 300193 China; 10 Department of Colorectal Surgery, Tianjin Union Medical Center, Nankai University, Tianjin, 300121 China

**Keywords:** Colorectal cancer (CRC), N6-methyladenosine (m^6^A), Ferroptosis, Fat mass and obesity-associated protein (FTO), Solute carrier family 7 member 11 (SLC7A11), Glutathione peroxidase 4 (GPX4)

## Abstract

Ferroptosis is a newly identified iron-dependent form of death that is becoming increasingly recognized as a promising avenue for cancer therapy. N6-methyladenosine (m^6^A) is the most abundant reversible methylation modification in mRNA contributing to tumorigenesis. However, the crucial role of m^6^A modification in regulating ferroptosis during colorectal cancer (CRC) tumorigenesis remains elusive. Herein, we find that m^6^A modification is increased during ferroptotic cell death and correlates with the decreased m^6^A demethylase fat mass and obesity-associated protein (FTO) expression. Functionally, we demonstrate that suppressing FTO significantly induces CRC ferroptotic cell death, as well as enhancing CRC cell sensitivity to ferroptosis inducer (Erastin and RSL3) treatment. Mechanistically, high FTO expression increased solute carrier family 7 member 11 (SLC7A11) or glutathione peroxidase 4 (GPX4) expressions in an m^6^A-YTHDF2 dependent manner, thereby counteracting ferroptotic cell death stress. In addition, we identify Mupirocin as a novel inhibitor of FTO, and Mupirocin induces CRC ferroptosis and inhibits tumor growth. Clinically, the levels of FTO, SLC7A11, and GPX4, are highly correlated expression in CRC tissues. Our findings reveal that FTO protects CRC from ferroptotic cell death in promoting CRC tumorigenesis through triggering SLC7A11/GPX4 expression.

**Supplementary Information:**

The online version contains supplementary material available at 10.1186/s13046-024-03032-9.

## Introduction

Colorectal cancer (CRC) is the third most common cancer in the world and has an increasing cancer incidence death worldwide [1]. Even with recent advances in diagnosis and therapy. However, the associated mortality rate remains high, mostly due to chemotherapy resistance in therapy process [2, 3]. Therefore, an understanding of the underlying molecular mechanism of CRC occurrence is of great significance for the development of more effective novel therapeutic approaches to eliminate and cure CRC.

Ferroptosis is a newly identified iron-dependent form of cell death, which is characterized by the accumulation of lipid peroxidation products malondialdehyde (MDA), intracellular iron accumulation, depletion of glutathione, and the decrease of glutathione peroxidase 4 (GPX4) activity and then results in lipid membrane damage and perforation [4–6]. More and more evidences show that inducing or inhibiting ferroptosis has great potential in treating cancer [4], as well as contribute to the efficacy of radiotherapy [7] and chemotherapy [8, 9]. Emerging evidence suggests chemotherapy drugs with ferroptosis inducer (Erastin or RSL3) resulted in a remarkable synergistic effect on tumor treatment [10, 11]. Furthermore, targeting solute carrier family 7 member 11 (SLC7A11) or GPX4 could specifically induce ferroptosis and suppress the progression of CRC [12, 13]. Therefore, exploring the mechanism of regulating the induction of ferroptosis is of great significance for developing effective treatment strategies for CRC.

N6-methyladenosine (m^6^A) is the most prevalent, abundant, and conserved internal chemical modification in mRNA [14], and m^6^A modification is reversible and catalyzed by corresponding enzymes, namely, “writers”, “erasers”, and “readers”. [15]. Emerging evidence suggests that the mRNA m^6^A modification plays oncogenic roles in various cancers and targets m^6^A modification presenting an opportunity for treating cancers [14, 16–18]. Recently, several studies have shown that m^6^A modification is involved in many physiological and pathological processes, including ferroptosis [19–23]. For example, Liu et al. found that m^6^A modification enhances ferroptosis resistance through inhibiting SLC7A11 mRNA deadenylation in hepatoblastoma [20]. Shen et al. reported that m^6^A modification-dependent ferroptosis as a potential target for the treatment of liver fibrosis [19]. However, the detailed roles and molecular mechanisms underlying m^6^A modification responds to ferroptotic cell death in CRC are not yet fully elucidated.

In the current study, we demonstrate that m^6^A modification is increased during ferroptotic cell death mediated by decreasing the expression of fat mass and obesity-associated protein (FTO), which down-regulates the expression of SLC7A11 and GPX4, thereby promoting ferroptosis in CRC. Furthermore, we develop a natural product Mupirocin as an inhibitor of FTO, which not only induces CRC ferroptosis, also show synergistic effect by combing Erastin or RSL3 on inhibiting the tumorigensis of CRC. Collectively, our results demonstrate how the mRNA m^6^A modification adds another dimension to the regulation of gene expression of ferroptosis cell death related genes in response to Erastin and RSL3 treatment.

## Materials and methods

The more detailed information of materials and methods are in supplementary information.

### Reagents and Biological resources

The Reagents and Biological Resources are listed in the key resources table of supplementary information.

### Cell culture

The colorectal cancer (CRC) cells were cultured in RPMI1640 (Thermo Fisher Scientific, MA, USA) supplemented with 10% fetal bovine serum (FBS, ExCell Bio, Shanghai, China). HEK 293T was cultured in Dulbecco’s modified Eagle’s medium (DMEM, Thermo Fisher Scientific, MA, USA) supplemented with 10% FBS, antibiotics, and 10 mM HEPES, and used for lenti-virus package.

### Cell proliferation assay

The CRC control and knocked down cells were seeded in 24-well plates. On the second day of seeding, the counting is started and lasts four days. CRC cells that were treated with inhibitors were seeded in 24-well plates. On the second day of seeding the cells, the appropriate concentration of inhibitor was added to the medium, and the cell count was started on the third day and continued for four days.

### Colony formation assay

The CRC control and knocked down cells or CRC cells treated with inhibitors were seeded in 6-well plates. Cells treated with inhibitors were added the appropriate concentration of inhibitor to the medium. Then the cells were continuous cultured for 1–2 weeks. Cells were stained by Crystal violet for counting. Colony number was measured by Image J.

### Molecular docking assay

Using Discovery Studio (DS) v19.1.0 software, we explored the accurate docking mode of Mupirocin and FTO (PDB: 3LFM). The SDF format files of the 3D structure of the Mupirocin were downloaded from the PunChem database (https://pubchem.ncbi.nlm.nih.gov/). The crystal structure of protein was retrieved from Protein Data Bank (PDB, https://www.rcsb.org/). At last, PyMOL software was used to present the results.

### Surface plasmon resonance (SPR) assay

The buffer was assembled according to the standard operating procedure of the BiacoreT200 instrument (GE healthcenter, USA) and the CM5 sensor chip was installed. After that, the purified protein was coupled to the chip, and then the surface of the chip was tested to select the appropriate concentration of different drug molecules for affinity experiment.

### In vitro Thermal shift assay

The same volume of TBS was added to two EP tubes, and the same amount of prokaryotic expressed protein was added. Mupirocin was added to one EP tube, and DMSO was added to the other EP tube. Incubating the mixed suspension for 25 min at 25 °C. Afterwards, the mixed suspension of each group were equally divided into PCR tubes, and then heat-shocked with different temperature gradients for 30 min. The signal was detected by Immobilon Western HRP Kit (Millipore, USA).

### mRNA stability assay

On day 1, shCtr group and shFTO group cells were inoculated into 12-well plates. After 24 h, the cells were cultured in fresh medium with a density of 70–80% for 4 h, and then Actinomycin D (5 µg/mL) was treated on the cells according to the time node of 0, 3, and 6 h, and the mRNA levels at each time point were analyzed by qPCR.

### In vitro m^6^A Demethylation assay base on cell-free system

Based on the published protocol, total RNA was isolated using TRIzol reagent, FTO proteins were purified from cells transfected with FTO by flag pull-down assay or purified from prokaryotic expression FTO protein by His-nickel column. The assay buffers include L-ascorbic (2 mM), HEPES (50 mM, PH = 7.0), (NH_4_)_2_Fe(SO_4_)_2_.6H_2_O (283 µM), αKG (300 µM), and BSA (50 µg/ml). Add RNAase H_2_O, buffers, RNA, proteins and inhibitor to 50 µL total system. Incubating for 3 h at room temperature, and then the Dot blot assay was conducted to quantify m^6^A.

### RNA immunoprecipitation (RIP) assay

Briefly, harvest and wash cells transfected with YTHDF1, YTHDF2, and YTHDF3, cells were lysed and incubated with Flag-beads overnight at 4 °C. Then washed the beads three times and Chloroform-isopropanol reagent was used to extract the RNA in the immunoprecipitates and inputs. The RT-qPCR was conducted to quantify SLC7A11 and GPX4 mRNA.

### m^6^A methylated RNA immunoprecipitation-qPCR (MeRIP-qPCR)

Briefly, total RNA was isolated and incubated with m^6^A antibody or normal rabbit IgG-conjugated Protein A/G Beads (Santa Cruz, CA, USA) in 500 µL buffer containing RNAase inhibitors overnight at 4 °C. RNA with m^6^A modifications were immunoprecipitated by m^6^A antibody-conjugated beads, washed 3 times and incubated with proteinase K digestion buffer. RNA was finally purified by Trizol/chloroform extraction and analyzed by RT-qPCR.

### RNA pulldown assay

Biotin-labelled RNA oligonucleotides were synthesized by the GENEWIZ (Suzhou, China). Single-stranded RNA baits were denatured at 99 °C for 10 min, after which they were immediately placed on ice. After that, 50 µL streptavidin magnetic beads (Thermo Fisher Scientific, MA, USA) were added to PBS buffer and incubated at 4 ° C for 4 h. RNA bait-conjugated streptavidin magnetic beads were then incubated with cell extracts in lysis buffer overnight at 4 ℃. After multiple washes, the RNA-protein complex was dissolved in 1× SDS buffer.

### Statistical analysis

Data were analyzed and mapped with GraphPad Prism 5 or GraphPad Prism 8, and were presented as mean ± SEM (standard error of mean) as indicated. Two-tailed Student’s t-test was used to compare means between groups as indicated and *p* < 0.05 was considered significant.

## Results

### m^6^A modification is increased during ferroptosis cell death in CRC cells

**Fig. 1:**
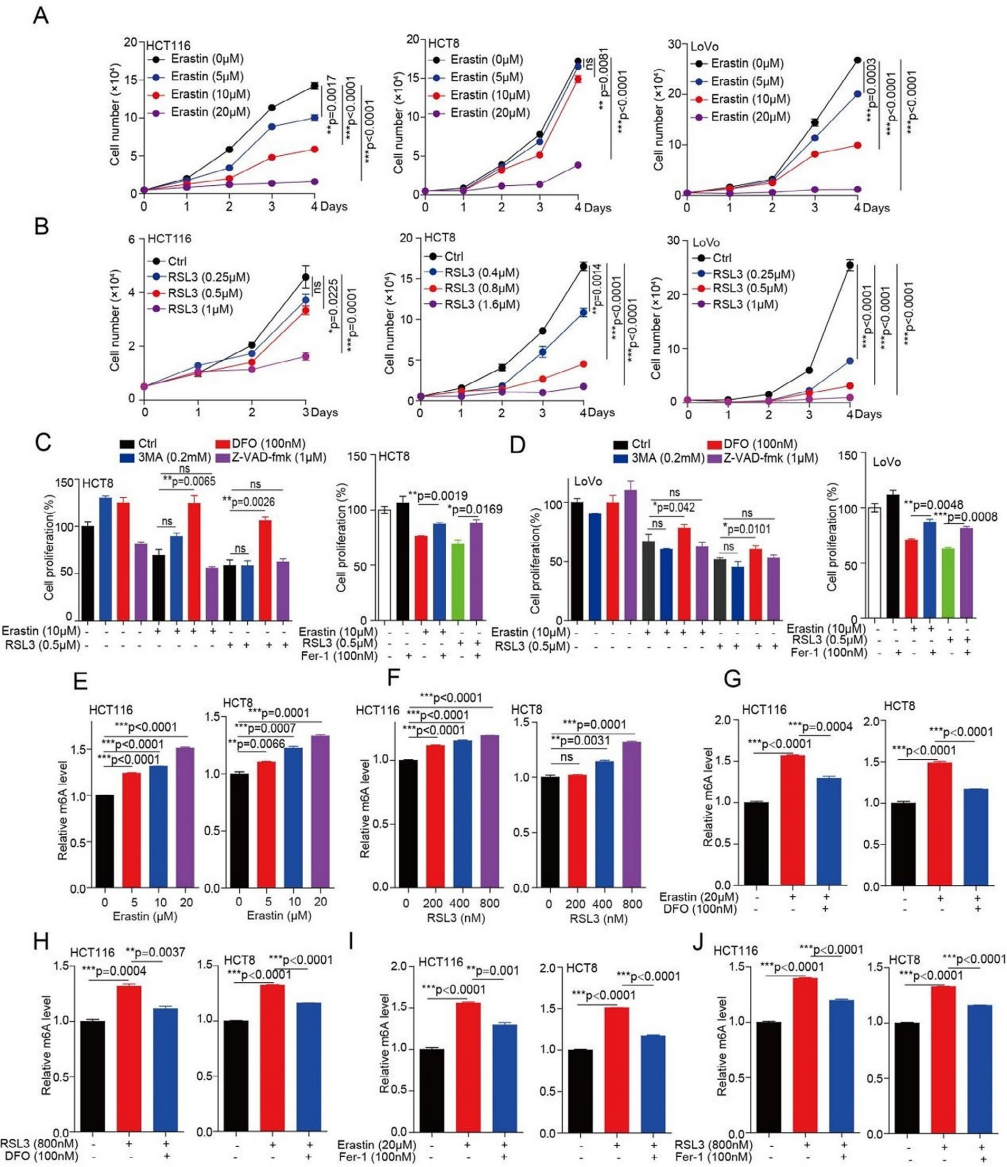
m^6^A modification is increased during ferroptosis cell death in CRC. (**A**-**B**) CRC cells treated with 5 µM, 10 µM, and 20 µM of Erastin (or indicate concentration of RSL3), and then harvested cells for counting cell number at indicated day 1, day 2, day 3, and day 4 to determine the cell proliferation. (**C**-**D**) CRC cells pr-treated with Erastin (or RSL3) for 4 h, subsequently treated with or without of DFO (DFO; 100 nM), 3MA (3MA; 0.2 mM), Z-VAD-FMK (Z-VAD; 1 µM), or ferrostatin1 (Fer-1; 100 nM) for another 72 h, and then harvested cells for counting cell number to determine the cell proliferation. (**E**-**F**) CRC cells treated with 5 µM, 10 µM, and 20 µM of Erastin (or 200 nM, 400 nM, and 800 nM of RSL3) for 72 h, and then the total RNA were harvested for ELISA to determine the m^6^A levels. (**G**-**H**) CRC cells treated with 20 µM of Erastin (or 800 nM of RSL3) for 4 h, subsequently treated with or without DFO (100 nM) for another 72 h, and then the total RNA were harvested for ELISA to determine the m^6^A levels. **(I**-**J**) CRC cells treated with 20 µM of Erastin (or 800 nM of RSL3) (4 h) in the absence or presence of Fer-1 (100 nM) for 72 h, and then the total RNA were harvested for ELISA to determine the m^6^A levels. (All error bars, mean values ± SEM (standard error of mean), p values were determined by unpaired two-tailed Student’s t test of *n* = 3 independent biological experiments. *p < 0.05; **p < 0.01; ***p < 0.001)

To investigate whether m^6^A modification is involved in ferroptosis during Erastin or RSL3 treatment, two well-established molecular compounds used as ferroptosis agonists. Firstly, we determined the IC50 of Erastin and RSL3 in inhibiting CRC cells, and found that Erastin and RSL3 have good inhibition in CRC cells at low concentration (Fig. S1a, b). Next, we explored the inhibition effect of Erastin and RSL3 on cell proliferation in CRC cells, and found that a significant inhibition on CRC cell proliferation by treating with either Erastin or RSL3 in a dose and time dependent manner (Fig. 1a, b and S1c, d). Lastly, we want to explore whether the inhibition effect of Erastin or RSL3 on CRC cell proliferation due to the ferroptotic death. Indeed, we found that the decreased cell proliferation could be prevented by simultaneously treating with iron chelator deferoxamine (DFO) or ferrostatin1 (Fer-1), not 3-Methyladenine (3-MA) and Z-VAD-fmk (Fig. 1c, d), suggesting that ferroptosis did occur during Erastin or RSL3 treatment in CRC cells.

To study whether ferroptosis poses effect on the m^6^A modification in CRC cells, we analyzed the effect of Erastin or RSL3 on m^6^A modification in CRC cells. Enzyme linked immunosorbent assay (ELISA) and m^6^A dot blotting assay both show that the m^6^A modification levels were increased in CRC cells by treating with either Erastin or RSL3 in a dose dependent manner (Fig. 1e, f and S1e, f). In addition, the increased m^6^A modification levels were prevented by treating with DFO or Fer-1 (Fig. 1g-j and S1g, h). Together, these findings suggested that Erastin or RSL3 treatment induce CRC ferroptotic cell death are associated with the increased m^6^A modification levels.

### m^6^A modification upregulation upon ferroptosis requires FTO

**Fig. 2:**
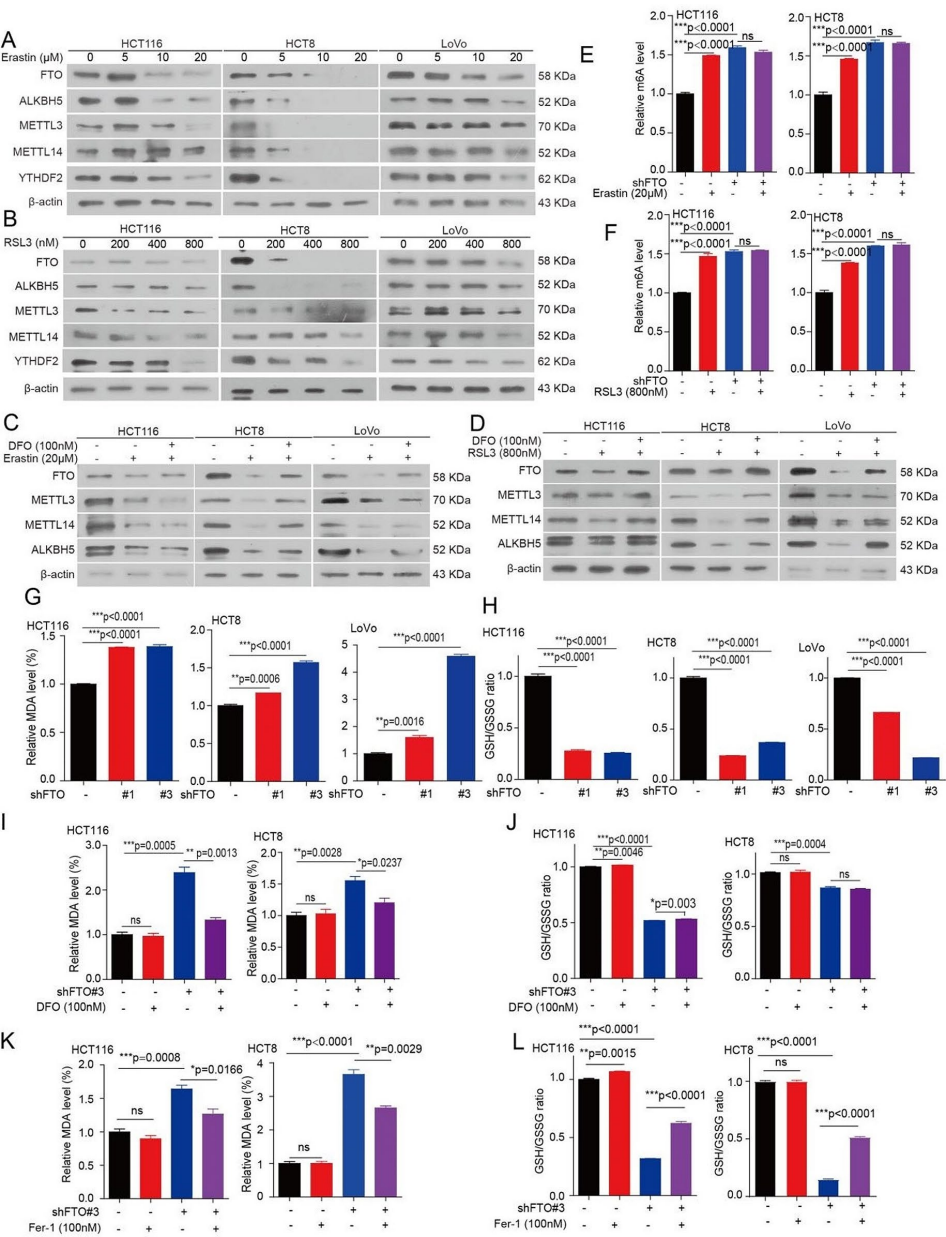
FTO mediates m^6^A modification upregulation during ferroptosis and regulates ferroptosis. (**A**-**B**) CRC cells treated with 5 µM, 10 µM, and 20 µM of Erastin (or 200 nM, 400 nM, and 800 nM of RSL3) for 72 h. The lysates were collected for western blotting to examine the expression of FTO, ALKBH5, METTL3, METTL14, and YTHDF2. (**C**-**D**) CRC cells pr-treated with 20 µM of Erastin (or 800 nM of RSL3) for 4 h, subsequently treated with or without of DFO (DFO; 100 nM) for 72 h. The lysates were collected for western blotting to examine the expression of FTO, ALKBH5, METTL3, and METTL14. (**E**-**F**) FTO knockdown or vector control CRC cells treated with 20 µM of Erastin (or 800 nM of RSL3) for 72 h, and then the total RNA were harvested for ELISA to determine the m^6^A levels. (**G**-**H**) The malondialdehyde (MDA) concentration or GSH/GSSG ratio were detected using assay kits in FTO knockdown or vector control CRC cells. (**I**-**J**) The MDA concentration or GSH/GSSG ratio were detected using assay kits in FTO knockdown or vector control CRC cells treated with or without 100 nM DFO for 72 h. (**K**-**L**) The MDA concentration or GSH/GSSG ratio were detected using assay kits in FTO knockdown or vector control CRC cells treated with or without 100 nM Fer-1 for 72 h. (All error bars, mean values ± SEM, p values were determined by unpaired two-tailed Student’s t test of *n* = 3 independent biological experiments. *p < 0.05; **p < 0.01; ***p < 0.001)

The formation of m^6^A modification is a reversible process, which added by m^6^A “writers”, inducing methyltransferase like METTL3, METTL14, and WTAP, and removed by FTO and alkB homolog 5 (ALKBH5) [14]. To understand how m^6^A modification upregulation during ferroptosis, we analyzed the effects of ferroptotic damage on the expression of FTO, ALKBH5, METTL3, METTL14, YTHDF2, and WTAP. We found that the mRNA levels of FTO, WTAP, and YTHDF2 were decreased with the increasing the concentration of Erastin or RSL3 in CRC cells, while the mRNA levels of ALKBH5, METLL3, and METLL14 were not significantly affected by Erastin or RSL3 (Fig. S2a, b). However, we found that the protein levels of FTO, ALKBH5, METTL3, METTL14, WTAP, and YTHDF2 were all decreased in CRC cells by treating with either Erastin or RSL3 in a dose dependent manner (Fig. 2a, b and S2c, d). In addition, the decreased FTO, ALKBH5, METTL3, METTL14, and WTAP levels were prevented by treating CRC cells with DFO or Fer-1 (Fig. 2c, d and S2e, h). Combined with the results from that the increased m^6^A modification levels have occurred during Erastin or RSL3 treatment in CRC cell. Thus, we assumed that m^6^A demethylase may be a driver for the increased m^6^A modification levels during ferroptosis. Next, we explore the determined which m^6^A demethylase regulates m^6^A modification during ferroptotic death, and found that m^6^A modification levels were still increased in knockdown ALKBH5 cells treated with Erastin or RSL3, however, the increased m^6^A modification levels in CRC cells treated with Erastin or RSL3 were blocked with the knockdown of FTO (Fig. 2e, f and S2i, l). Furthermore, we found that knockdown of FTO decreased the levels of ALKBH5, METTL3, and METTL14 (Fig. S2m), which explained the decreased levels of ALKBH5, METTL3, METTL14, and WTAP were mediated by FTO during the CRC cells ferroptotic death. Collectively, these results demonstrated FTO is responsible for the m^6^A modification change during ferroptosis.

### FTO mediates CRC cells ferroptosis

Given that FTO expression is downregulated during CRC ferroptotic cell death, we assumed that FTO may be a negative regulator of ferroptosis. Indeed, we found that knockdown FTO augmented MDA levels, and decreased the glutathione/glutathione (GSH/GSSG) ratio (Fig. 2g, h). Furthermore, targeting FTO by Rhein also decreased GSH/GSSG ratio and the increased MDA levels (Fig. S2n, o). While, the increased MDA levels and decreased GSH/GSSG ratio were not only prevented by treating with DFO or Fer-1 in the FTO knockdown cells (Fig. 2i-l), but also can be rescued by exogenous expression FTO (synonymous mutation, with a shRNA-resistant FTO) in FTO knockdown cells (Fig. S2p, q). In addition, the decreased GSH/GSSG ratio was also prevented by treating with glutathione (GSH) (Fig. S2r). Collectively, these findings suggested that FTO protects CRC cells from ferroptotic cell death.

### Targeting FTO sensitizes CRC cells to ferroptosis

**Fig. 3:**
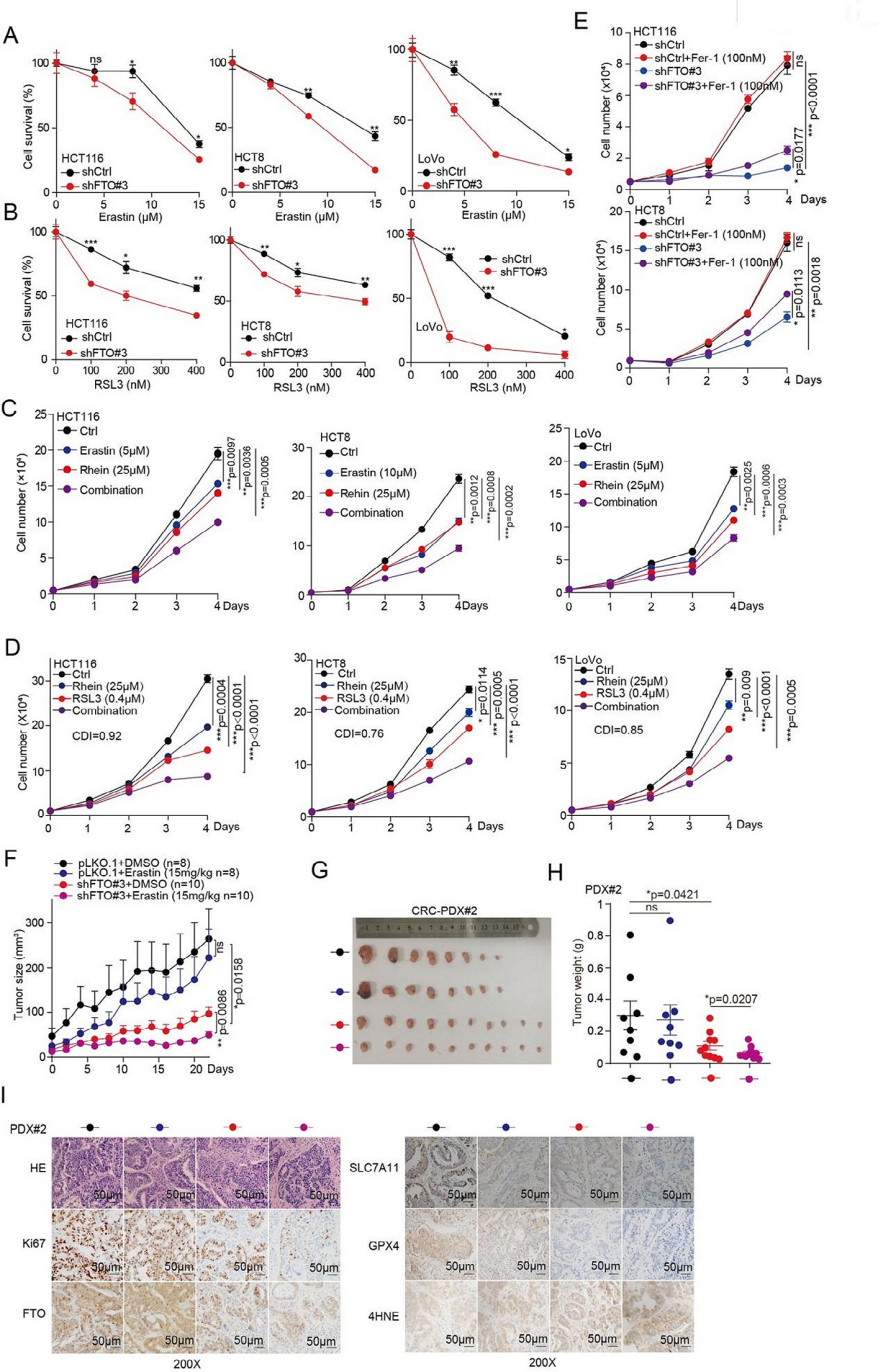
Targeting FTO enhances the anti-tumor effects of Erastin and RSL3. (**A**-**B**) Cell survival was determined in CRC cells with stable FTO knockdown when treated with 0 µM, 5 µM, 10 µM and 15 µM of Erastin (or 0 nM, 100 nM, 200 nM, 300 nM, and 400 nM of RSL3), and then harvested cells for counting cell number at indicated day 3. (**C**-**D**) CRC cells treated with 25 µM Rehin, 5 µM of Erastin (or 400 nM of RSL3), alone or in combination, and then and then harvested cells for counting cell number at indicated day 1, day 2, day 3, and day 4 to determine the cell proliferation. (**E**) Cell proliferation was determined in CRC cells with stable FTO knockdown when treated with or without 100 nM of Fer-1, and then harvested cells for counting cell number at indicated day 3. (**F**) Tumor growth was compared between xenograft nude mice bearing with CRC PDX injected with FTO shRNA virus and control shRNA virus IP injection with or without Erastin (15 mg/kg per two days). Tumor volume was calculated for each group at the indicated times. (**G**) All tumors from nude mouse are shown. (**H**) Tumor mass in xenograft nude mice bearing with CRC PDX injected with FTO shRNA virus and control shRNA virus IP injection with or without Erastin (15 mg/kg per two days). (**I**) HE, Ki67, FTO, SLC7A11, GPX4, and 4HNE were analyzed in a representative PDX xenograft tumor by IHC (scale bar = 50 μm). (All error bars, mean values ± SEM, p values were determined by unpaired two-tailed Student’s t test of *n* = 3 independent biological experiments. *p < 0.05; **p < 0.01; ***p < 0.001)

Our previous studies showed that FTO protects CRC cells from ferroptotic cell death, thus, we wonder whether pharmacological blocking of FTO would as a potential therapeutic approach in cancer with a ferroptotic cell death. We found that knockdown FTO increased the sensitivity of CRC cells to the Erastin or RSL3 treatment (Fig. 3a, b). Next, we hypothesized that a combination of FTO inhibitor and Erastin or RSL3 would show synergistic effects. Indeed, targeting FTO by Rhein increased the anticancer of Erastin or RSL3 in CRC cells (Fig. 3c, d and S3a, b). However, as DFO and Fer-1 have been marketed as ferroptosis inhibitor, which against the inhibition of targeting FTO on cell proliferation with a ferroptotic signature (Fig. 3e and S3c). Thus, these results indicated that a key role of FTO expression or activity in inhibiting the anticancer activity with a ferroptosis signature.

As the expression of METTL3, METTL14, and ALKBH5 were decreased under Erastin or RSL3 treatment mediated by FTO, we also examined the response of knockdown METTL3, METTL14, and ALKBH5 to Erastin or RSL3 treatment in CRC cells. The results showed that knockdown of METTL3 or METTL14 decreased the sensitivity of CRC cells to the Erastin or RSL3 treatment (Fig. S3d, e). While, knockdown of ALKBH5 increased the sensitivity of CRC cells to the Erastin or RSL3 treatment (Fig. S3f, g). These results suggested that METTL3, METTL14, and ALKBH5 are also involved the CRC ferroptosis induced by Erastin or RSL3.

We next sought to determine whether the genetic inhibition of FTO can enhance the in vivo anticancer activity to induce ferroptosis. We first examined the effect of FTO on tumor growth, then, we established a patient-derived xenograft (PDX) model, and found that knockdown FTO significantly decreased the tumor growth (Fig. S3h, i), tumor masses (Fig. S3j), and Ki67 expression, as well as with the increased ferroptosis biomarker 4-hydroxynonenal (4HNE) levels (Fig. S3k). Next, we used this PDX model, and explored the effect of inhibition of FTO on Erastin treatment. We found that Erastin has a slight inhibitory effect on the tumor growth in FTO knockdown control group (Fig. 3f, g). While the administration of Erastin at 15 mg/kg in FTO knockdown group significantly suppressed the xenograft tumor growth (Fig. 3f, g), tumor masses (Fig. 3h), and Ki67 expression (Fig. 3i). Interesting, the inhibition of FTO expression conferred Erastin therapy sensitivity that was associated with increased ferroptosis biomarker 4HNE levels (Fig. 3i). These preclinical animal studies support the hypothesis that targeting FTO significantly enhances the anticancer activity of Erastin in vivo.

### FTO enhances the expression of SLC7A11 and GPX4 in CRC cells

**Fig. 4:**
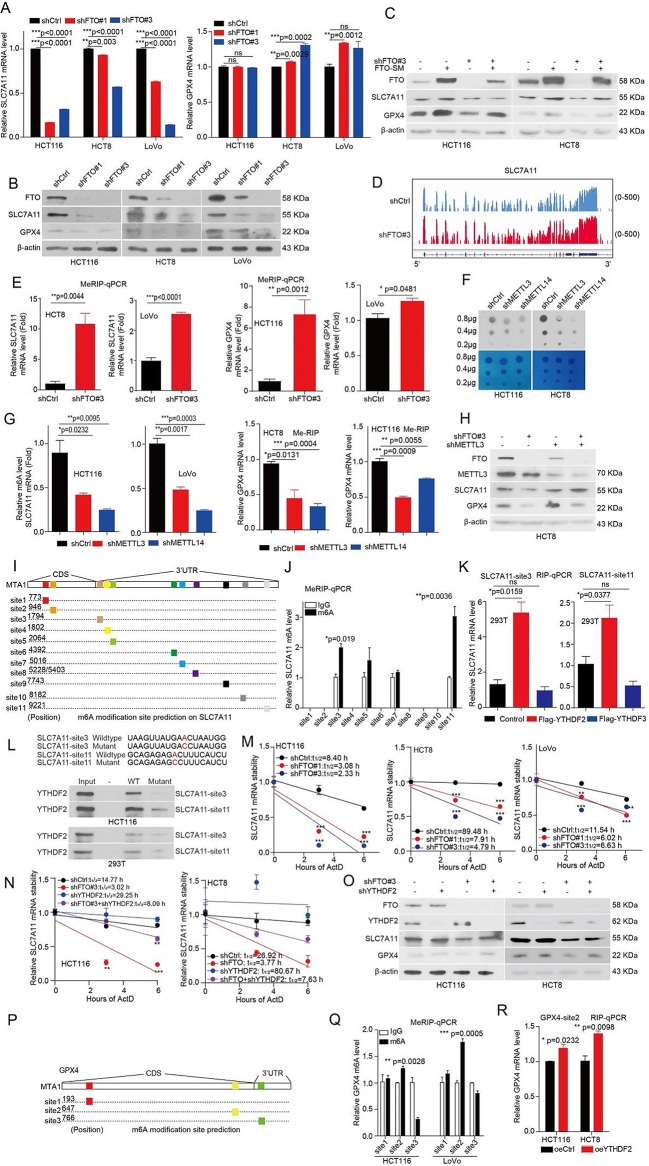
FTO enhances the expression of SLC7A11 and GPX4 in CRC cells. (**A**)The total RNA were extracted from FTO knockdown or vector control CRC cells, and then mRNA was reversely transcribed into cDNA. The expression of SLC7A11 and GPX4 mRNA were examined by qRT-PCR. (**B) **The lysates were collected from FTO knockdown or vector control CRC cells for western blotting to examine the expression of FTO, SLC7A11, and GPX4. (**C**) FTO knockdown HCT116 and HCT8 cells with or without exogenous expression of FTO. The lysates were collected for western blotting to examine the expression of FTO, SLC7A11, and GPX4. (**D**) The relative abundance of m^6^A sites along SLC7A11 mRNA in FTO knockdown cells and control cells, as detected by m^6^A-seq. (**E**) The m^6^A modification levels on SLC7A11/GPX4 were examined by the MeRIP-qPCR in FTO knockdown cells and control CRC cells. (**F**) The total RNA were harvested from METTL3 or METTL14 knockdown and control cells, then the total RNA for dot blotting assay to determine the m^6^A levels. (**G**) The m^6^A modification levels on SLC7A11/GPX4 were examined by the MeRIP-qPCR in METTL3 or METTL14 knockdown cells and control cells (**H**) The lysates were collected from FTO knockdown or vector control CRC cells with or without knockdown METTL3 for western blotting to examine the expression of FTO, METTL3, SLC7A11, and GPX4. (**I**) Schematic diagram of SLC7A11 mRNA and the predicted ‘m^6^A’ sites at CDS and 3’UTR are highlighted. (**J**) Measurement of the m^6^A modification on eleven m^6^A-site clusters of SLC7A11 by the MeRIP-qPCR. (**K**) RIP-qPCR analysis to screen the reader protein by binding SLC7A11 mRNA. (**L**) Immunoblotting of YTHDF2 in HCT116 and 293T cells was pull downed by biotinylated-SLC7A11 (site 3 and site11) and the biotinylated-SLC7A11 (site 3 and site11) without m^6^A motif mutation. (**M**) FTO knockdown or vector control CRC cells were treated with 5 µg/mL actinomycin D (Actd) as indicated and were subjected to qRT-PCR analysis for the mRNA stability of SLC7A11. (**N**) FTO knockdown or vector control CRC cells with or without knockdown YTHDF2, and then treated with 5 µg/mL actinomycin D (Actd) as indicated and were subjected to qRT-PCR analysis for the mRNA stability of SLC7A11. (**O**) CRC cells with or without knockdown YTHDF2. The lysates were collected for western blotting to examine the expression of FTO, YTHDF2, and SLC7A11. (**P**) Schematic diagram of SLC7A11 mRNA and the predicted ‘m^6^A’ sites at CDS and 3’UTR are highlighted. (**Q**)Measurement of the m^6^A modification on three m^6^A-site clusters of GPX4 by the MeRIP-qPCR. (**R**) RIP-qPCR analysis to screen the reader protein by binding SLC7A11 mRNA. (All error bars, mean values ± SEM, p values were determined by unpaired two-tailed Student’s t test of *n* = 3 independent biological experiments. *p < 0.05; **p < 0.01; ***p < 0.001)

To explore the molecular mechanism of FTO in regulating ferroptotic cell death, the RNA-sequencing (RNA-Seq) was performed in FTO knockdown and vector control cells. A total of 1, 655 significant differentially expressed genes were observed in FTO knockdown cells (Fig. S4a). What is more, heatmap analysis indicated a clear separation between the ferroptotic related gene expression profiles of FTO knockdown and vector control cells (Fig. S4b). As given that knockdown FTO enhances the anti-tumor effects of Erastin and RSL3, which targets SLC7A11 and GPX4, respectively. Thus, we next focus on the SLC7A11 and GPX4 as downstream of FTO in regulating ferroptosis. Validation studies showed that knockdown FTO significantly decreased mRNA levels of SLC7A11, while increased mRNA levels of GPX4 in CRC cells (Fig. 4a), these results are consistent with the RNA-seq data. Furthermore, we found that knockdown FTO significantly decreased protein levels of both SLC7A11 and GPX4 (Fig. 4b), while, a shRNA-resistant FTO can rescued the decreased protein levels of SLC7A11 and GPX4 in the FTO knockdown cells (Fig. 4c). Taken together, these results suggested that FTO regulates SLC7A11 and GPX4 expression.

### FTO-mediated m^6^A modification enhances the expression of SLC7A11 mRNA

To explore the function of FTO in regulating SLC7A11 or GPX4 expression whether dependent on FTO m^6^A demethylase activity, we analyzed our published methylated RNA immunoprecipitation-m^6^A-sequencing (MeRIP-m^6^A-seq) data [18] and found that the m^6^A peaks were increased on SLC7A11 (Fig. 4d) and GPX4 mRNA (Fig. S4c) in FTO knockdown group. To validate the MeRIP-m^6^A-seq data, we applied the methylated RNA immunoprecipitation qPCR (MeRIP-qPCR) assay to confirm that SLC7A11 mRNA and GPX4 mRNA have m^6^A modification (Fig. S4d, e). Furthermore, we found that knockdown of FTO significantly increased the m^6^A modification levels on SLC7A11 mRNA and GPX4 mRNA in CRC cells (Fig. 4e). The m^6^A modification is a dynamically reversible process, added by methyltransferases (Writers: METTL3, METTL14) and removed by demethylases (Erasers: FTO, ALKBH5) [15]. Here, we generated METTL3 and METTL14 knockdown cells with the decreased total m^6^A levels (Fig. 4f) and found that METTL3 and METTL14 are responsible for the m^6^A modification on SLC7A11 mRNA and GPX4 mRNA (Fig. 4g). Besides, METTL3 can also rescued the decreased protein levels of SLC7A11 and GPX4 in the FTO knockdown cells (Fig. 4h). These results suggested that FTO regulates SLC7A11 and GPX4 expression mediated by m^6^A modification and can by reversed by its methyltransferase METTL3.

### FTO enhances the expression of SLC7A11/GPX4 mediated by YTHDF2

As the m^6^A modification is recognized and bound by m^6^A-binding proteins (Readers), such as, YTH family, which play a specific role in control the fate of the methylated mRNA [15]. Next, we performed a RNA immunoprecipitation qPCR (RIP-qPCR) assays to screen for SLC7A11-related m^6^A readers and explore the direct interaction between the YTHDF1, YTHDF2, or YTHDF3 and SLC7A11 mRNA. RIP-qPCR assays showed that YTHDF2 and YTHDF3 bound to SLC7A11 mRNA (Fig. S4f), while only YTHDF2 bound to GPX4 mRNA (Fig. S4g). Next, we explore the exactly sites on SLC7A11 mRNA were recognized by YTHDF2 or by YTHDF3, and found there are have 11 potential m^6^A modifications by SRAMP analysis (www.cuilab.cn/sramp) (Fig. 4i). First, we validated that m^6^A modification site 3 and site 11 were predominantly m^6^A modification (Fig. 4j). Next, we found that the m^6^A modification site 3 and site 11 were recognized and bound by YTHDF2 not YTHDF3 (Fig. 4k). Lastly, we performed a streptavidin RNA pull-down assay further verified that YTHDF2 not YTHDF3 predominantly bound to the site 3 and site 11 on SLC7A11 mRNA (Fig. 4l). As YTHDF2 mediates the degradation of mRNA [24]. Thus, we next focus on the function of YTHDF2 in regulating SLC7A11 mRNA stability. Indeed, we found that the SLC7A11 mRNA stability was markedly decreased upon FTO knockdown in CRC cells (Fig. 4m). While, the decreased SLC7A11 mRNA stability and SLC7A11 protein levels were rescued by knockdown YTHDF2 in FTO loss cells (Fig. 4n, o). Taken together, our data suggested that FTO enhances the expression of SLC7A11 in a m^6^A modification mediated by YTHDF2.

Next, we explore the exactly sites on GPX4 mRNA were recognized by YTHDF2, and found there are 3 potential m^6^A modifications by SRAMP analysis (www.cuilab.cn/sramp) (Fig. 4p). Firstly, we validated that m^6^A modification site 2 was predominantly m^6^A modification (Fig. 4q). Secondly, we found that the m^6^A modification site 2 on GPX4 mRNA was recognized and bound by YTHDF2 (Fig. 4r). Our previously study showed that YTHDF2 promote 6-phosphogluconate dehydrogenase (6PGD) mRNA translation [25]. Then, we constructed pmirGLO-GPX4-site 2 m^6^A wild type and site 2 m^6^A mutant plasmids. The reporter mRNA translation assay showed that YTHDF2 promotes the GPX4 mRNA m^6^A wild type translation not m^6^A mutant (Fig. S4j). Thus, our data suggested that FTO enhances the expression of GPX4 in a m^6^A modification mediated by YTHDF2, which facilitates GPX4 mRNA translation.

As DFO and Fer-1 can rescue the decreased FTO expression under Erastin or RSL3 treated condition. Thus, we wonder whether DFO and Fer-1 also rescue the decreased SLC7A11 and GPX4 expression under Erastin or RSL3 treated condition. Indeed, the decreased SLC7A11 and GPX4 expression under Erastin or RSL3 treated condition were rescued by treating cells with DFO and Fer-1 (Fig. S4h,i). These results suggested that the expression of GPX4 and SLC7A11 were decreased by Erastin or RSL3 treatment mediated by FTO in a YTHDF2-denpendent manner.

### FTO regulates ferroptosis and cell proliferation via SLC7A11/GPX4

**Fig. 5:**
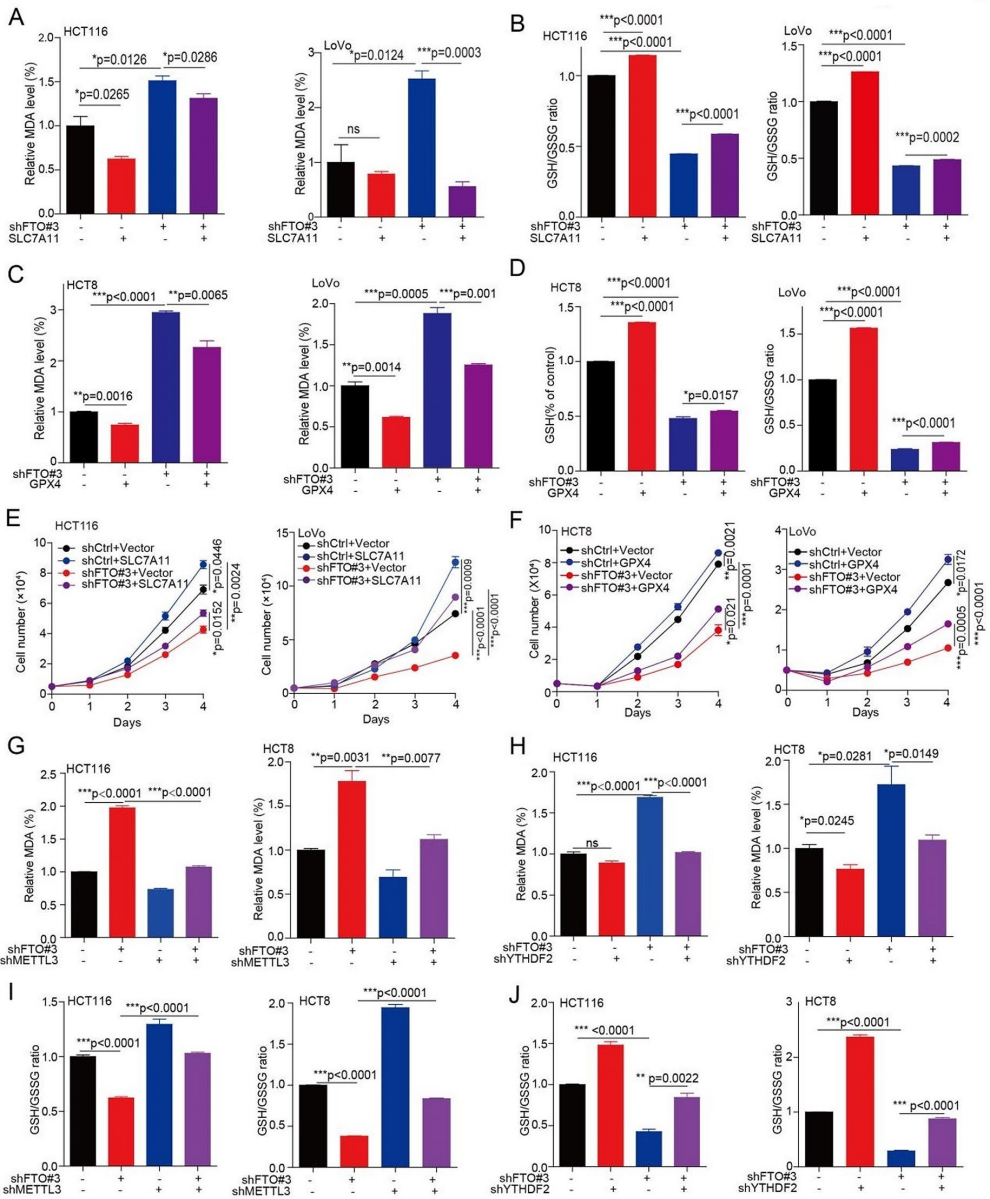
FTO regulates ferroptosis and cell proliferation via SLC7A11/GPX4. (**A**-**B**) The MDA concentration or GSH/GSSG ratio were detected using assay kits in FTO knockdown or vector control CRC cells with or without exogenous expression of SLC7A11. (**C**-**D**) The MDA concentration or GSH/GSSG ratio were detected using assay kits in FTO knockdown or vector control CRC cells treated with or without exogenous expression of GPX4. (**E**-**F**) The cell proliferation was determined in FTO knockdown or vector control CRC cells with or without exogenous expression of SLC7A11 (or GPX4), and then harvested cells for counting cell number at indicated day 1, day 2, day 3, and day 4 to determine the cell proliferation. (**G**-**H**) The MDA concentration were detected using MDA assay kits in FTO knockdown or vector control CRC cells with or without knockdown METTL3 (or YTHDF2). (**I**-**J**) The GSH/GSSG ratio was detected using GSH/GSSG assay kits in FTO knockdown or vector control CRC cells with or without knockdown METTL3 (or YTHDF2). (All error bars, mean values ± SEM, p values were determined by unpaired two-tailed Student’s t test of *n* = 3 independent biological experiments. *p < 0.05; **p < 0.01; ***p < 0.001)

To determine whether FTO regulates ferroptosis and cell proliferation mediated by regulating SLC7A11/GPX4 expression. We then forced expression of SLC7A11 or GPX4 into FTO knockdown cells. Firstly, we found that the exogenous expression of SLC7A11 in FTO knockdown cells rescued the increased MDA level and the decreased GSH/GSSG ratio (Fig. 5a, b and S5a). Similarly, the exogenous expression of GPX4, in FTO knockdown cells rescued the increased MDA level and the decreased GSH/GSSG ratio (Fig. 5c, d and S5b). Lastly, the decreased cell proliferation in FTO knockdown cells was rescued by forced expression of SLC7A11 or GPX4 (Fig. 5e, f and S5c). Taken together, these data suggested that FTO promotes cell proliferation via inhibiting ferroptotic cell death mediated by SLC7A11/GPX4.

As METTL3 and METTL14 are responsible for the m^6^A modification on SLC7A11/GPX4 mRNA, and the m^6^A modification on SLC7A11 mRNA is recognized by YTHDF2. Thus, we explore the role of METTL3, METL14, and YTHDF2 in regulating ferroptosis under the FTO loss condition. We found that knockdown METTL3 or METTL14 decreased product of MDA (Fig. S5d), and knockdown METTL3 not METTL14 increased the glutathione/glutathione (GSH/GSSG) ratio (Fig. S5e). Then, we further knockdown the expression of METTL3 or YTHDF2 in FTO knockdown cells, and found that knockdown of METTL3 or YTHDF2 rescued the increased MDA level in FTO loss condition (Fig. 5g, h). Lastly, knockdown of METTL3 or YTHDF2 also rescued the decreased GSH/GSSG ratio (Fig. 5i, j). While, when knockdown METTL3 or YTHDF2, the regulation of Erastin or RSL3 on MDA and GSH/GSSG ratio were abolished (Fig. S5f, g). These results suggested that FTO regulates ferroptosis by control SLC7A11 mRNA mediated by its m^6^A modification.

#### Discovery of a novel and potent FTO inhibitor Mupirocin

**Fig. 6:**
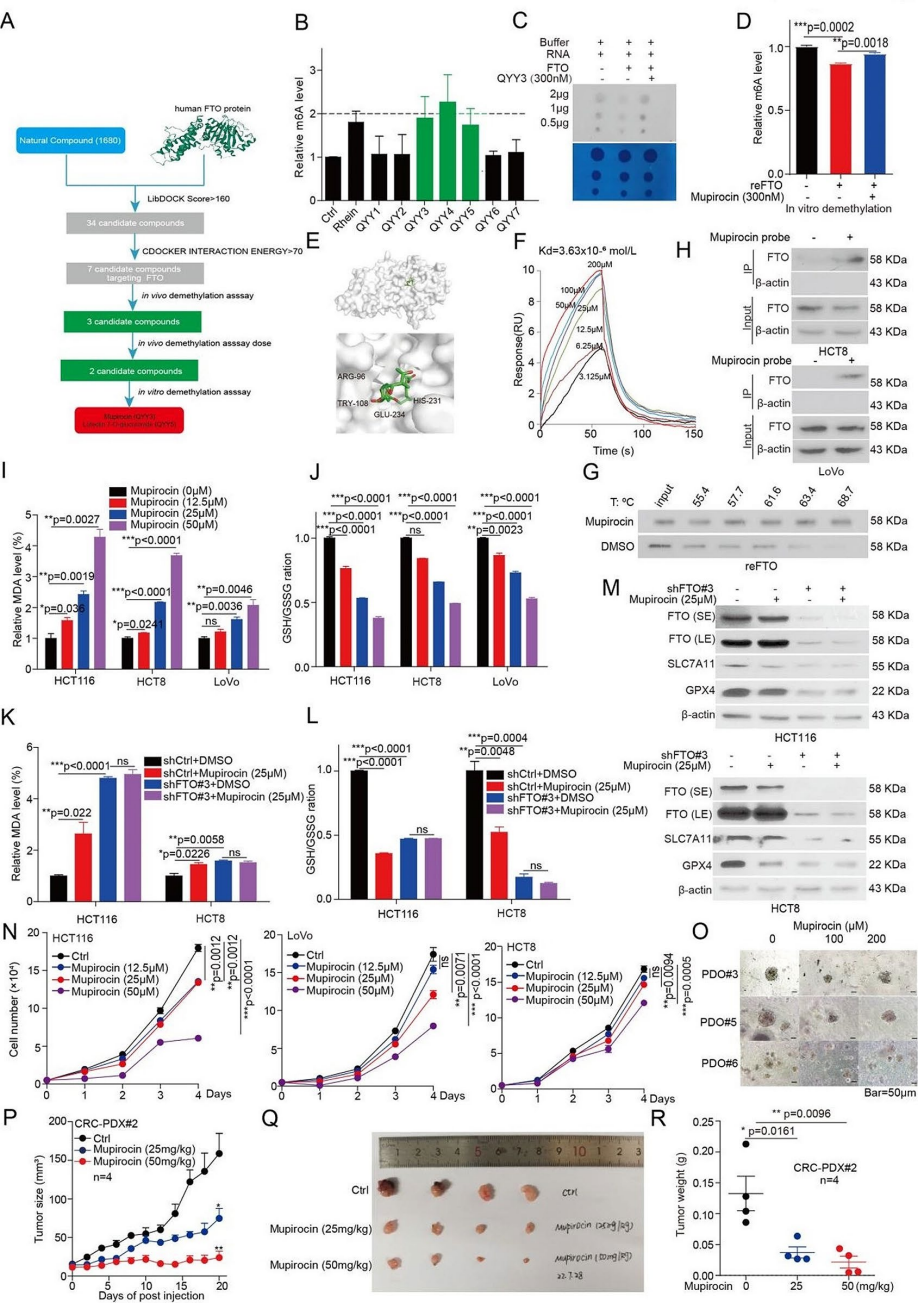
Identification of Mupirocin as a novel inhibitor of FTO and regulates CRC ferroptosis and tumor growth. (**A**) The flowchart of the pipeline to identify FTO inhibitors based on virtual screening. (**B**) HCT116 cells were treated seven compounds, and then the total RNA were harvested for dot blotting assay to determine the m^6^A levels. The optical density of blotting bands was quantified by Image J software and normalized to control. (**C)** The total RNA were harvested from HCT116 cells, and then incubated with FTO protein from HCT116 cells by flag pull-down in kinase buffer with or without Mupirocin. The enzymatic activity of FTO was anlysis by dot blotting assay. (**D**) The total RNA were harvested from HCT116 cells, and then incubated with recombinant FTO protein in kinase buffer with or without Mupirocin. The enzymatic activity of FTO was anlysis by ELISA. (**E**) The binding model of Mupirocin in FTO catalytic pocket. (**F**) The affinity of Mupirocin (0, 3.125, 6.25, 12.5, 25 µM, 50 µM, 100µM,  and 200 µM) for FTO was determined using SPR. (**G**) Thermal shift analysis for the affinity of Mupirocin for FTO, and then anlyzed by western blotting. (**H**) The in situ pull-down assay in CRC cells was performed to identifying the interaction between Mupirocin probe and FTO proteins. (**I**-**J**) The MDA concentration or GSH/GSSG ratio were detected using assay kits in CRC cells treated with the indicated doses of Mupirocin for 72 h. (**K**-**L**) The MDA concentration or GSH/GSSG ratio were detected using assay kits in FTO knockdown CRC cells treated with the indicated doses of Mupirocin for 72 h. (**M**) The expression of FTO, SLC7A11, and GPX4 were examined by western blotting in FTO knockdown CRC cells treated with the indicated doses of Mupirocin for 72 h. (**N**) CRC cells treated with 12.5 µM, 25 µM, and 50 µM of Mupirocin, and then harvested cells for counting cell number at indicated day 1, day 2, day 3, and day 4 to determine the cell proliferation. (**O**) CRC organoids treated with 100 µM and 200 µM of Mupirocin for 7 days, and then the pictures were taken for examining the effect of Mupirocin on growth of CRC organoids. Representative images of organoids treated with the indicated doses of Mupirocin (scale bar = 50 μm). (**P**) Tumor growth was compared between xenograft nude mice bearing with CRC PDX, whch IP injection with 25 mg/kg and 50 mg/kg Mupirocin (*n* = 4). Tumor volume was calculated for each group at the indicated times. (**Q**) All tumors from nude mouse were shown. (**R**) Tumor mass of xenograft nude micewith PDX tumor treated with Mupirocin. (All error bars, mean values ± SEM, p values were determined by unpaired two-tailed Student’s t test of *n* = 3 independent biological experiments. *p < 0.05; **p < 0.01; ***p < 0.001).

To identify potential FTO inhibitors, which suppress CRC tumor growth by inducing ferroptosis, we conducted a structure-based virtual screening of a small molecule library consisting of 1680 bioactive compounds based on the FTO crystal structure (PDB code: 3LFM). Firstly, 34 candidate compounds were appeared, when we set the the LibDOCK Score > 160. Then, 7 candidate compounds showed the highest docking scores with FTO by improving docking results (Fig. 6a and S6a). To validate the molecular docking results. Firstly, we performed in vivo demethylation assay. Then we assessed their efficacy on inhibition of FTO’s m^6^A demethylase activity, and identified three compounds (Mupirocin, Vitexin-4-O-glucoside, and Luteolin 7-O-glucuronide) have the best efficacy activity on inhibition of FTO’s m^6^A demethylase activity *in vivo* (Fig. 6b). In addition, cell-free m^6^A demethylase assays showed that Mupirocin and Luteolin 7-O-glucuronide exert effects on inhibition of FTO’s demethylase activity in vitro (Fig. 6c, d and S6b). As Mupirocin has best activity against FTO both in vivo and in vitro. Especially, it has not been reported to exert any anti-tumor effect in CRC, thus, we decided to focus on Mupirocin as novel inhibitor of FTO for further studies.

The molecular docking study based on the crystal structure of FTO (PDB code: 3LFM) suggested that Mupirocin fits in a catalytic pocket [26], surrounded by residues including R96, Y108, H231, and E234 of FTO (Fig. 6e). To examine whether Mupirocin binds to and inhibits FTO, we then performed Surface Plasmon Resonance (SPR) assays to determine affinity between Mupirocin and FTO protein, with a Kd at 3.63 × 10^− 6^ mol/L (Fig. 6f). Additionally, an *in vitro* thermal shift assay showed that the stability of the purified FTO was increased under the increasing concentrations of Mupirocin relative to DMSO treated group (Fig. 6g). According to the docking poses of Mupirocin and FTO protein, residues R96, Y108, H231, and E234 are essential for the binding of FTO with Mupirocin. To further evaluate Mupirocin target engagement, we performed a cellular thermal shift assay (CETSA) to examine the direct interaction of FTO and Mupirocin in LoVo cells, which transfected with Flag tag FTO WT, H231A/D233A, and R96A. CETSA assay showed that Mupirocin could block temperature-induced of wild-type (WT) FTO, but not that of mutant FTO H231A/D233A or FTO R96A (Fig. S6c). In summary, these results suggested that Mupirocin directly binds to the FTO.

To further analyze the interaction of Mupirocin with FTO, we synthesized a Mupirocin probe (Fig. S6d). The cell-free m^6^A demethylase assay also showed that Mupirocin probe exerts directly inhibitory effects on FTO’s demethylase activity in vitro (Fig. S6e, f). Furthermore, the anti-tumor activity showed that Mupirocin probe showed a comparable IC50 value with that of Mupirocin (Fig. S6g). Moreover, the in situ pull-down assay was performed and showed that Mupirocin could bind with FTO directly in HCT8 and LoVo cells (Fig. 6h). In all, such data confirmed that FTO binds directly with Mupirocin in the native cellular environment, and the mutated amino acids are essential for their interactions.

### Mupirocin treatments modulate the signaling pathways of FTO

To explore the role and molecular mechanisms underlying effects of Mupirocin by targeting FTO, RNA-Seq was performed in Mupirocin treated samples and control groups. A total of 2,347 significant differentially expressed genes were observed in Mupirocin treated samples (Fig. S6h). Through global gene set enrichment analysis (GSEA) [27], we identified a set of up-regulated or down-regulated pathways upon Mupirocin treatment, or FTO knockdown. Notably, among the up-regulated or down-regulated pathways, Mupirocin treatment, and FTO knockdown groups shared the majority of their enriched signaling pathways, such as G2M checkpoint, E2F target, apoptosis, MYC target V1, and ferroptosis (Fig. S6i). What is more, heatmap analysis indicated a clear separation between the ferroptotic related gene expression profiles of Mupirocin treatment and vector control cells as FTO knockdown RNA-Seq (Fig. S6j). Taken together, these results suggested that a novel and potent FTO inhibitor Mupirocin has similar effect as FTO in regulating CRC function.

#### Mupirocin triggers ferroptosis and suppresses tumor growth in CRC

The above results suggested that Mupirocin serve as a novel and potent FTO inhibitor. Therefore, we next assumed whether Mupirocin regulates CRC ferroptosis and tumor growth through targeting FTO. Firstly, it was found that the Mupirocin treatment resulted in an increased MDA levels (Fig. 6i), and the decreased GSH/GSSG ratio (Fig. 6j). While, the effect of Mupirocin on MDA levels and GSH/GSSG ratio were blocked by knockdown of FTO (Fig. 6k, l). Lastly, western blotting showed that Mupirocin treatment resulted in decreasing the expression of SLC7A11 and GPX4 protein (Fig. S6k). Again, the effect of Mupirocin on the expression of SLC7A11 and GPX4 protein were also blocked by knocking down FTO (Fig. 6m). These results suggested that Mupirocin treatment could trigger ferroptosis in CRC cells by targeting FTO.

To examine the effects of Mupirocin on CRC cell proliferation, CRC cells were treated with various concentrations of Mupirocin, and the effect of Mupirocin on cell proliferation was determined. Cell proliferation and colony formation assays showed that Mupirocin exerted a strong inhibition efficacy on CRC cell proliferation in a time and dose-dependent manner (Fig. 6n and S6l). Again, a similar inhibitory effect was also observed in the CRC patient-derived organoid (PDO) model with indicated doses of Mupirocin (Fig. 6o and S6m). These data demonstrated that Mupirocin has a significant inhibitory effect on CRC cell proliferation *in vitro*.

Next, the anti-tumor effects of Mupirocin were determined *in vivo* using a PDX mouse model. Then, 25 and 50 mg/kg of Mupirocin was administrated intraperitoneal (ip) every two days into PDX mouse model. Mupirocin significantly decreased the tumor growth (Fig. 6p, q), tumor masses (Fig. 6r), Ki67 expression, GPX4, and SLC7A11 levels, as well as induced the expression of ferroptosis biomarker 4HNE (Fig. S6n). There was no significant difference in body weight between the drug treatment group and the control group (Fig. S6o). These data suggested that Mupirocin significantly suppresses excessive tumor growth in vivo by targeting FTO through inducing ferroptosis.

#### Mupirocin enhances the anti-tumor effects of Erastin and RSL3

**Fig. 7:**
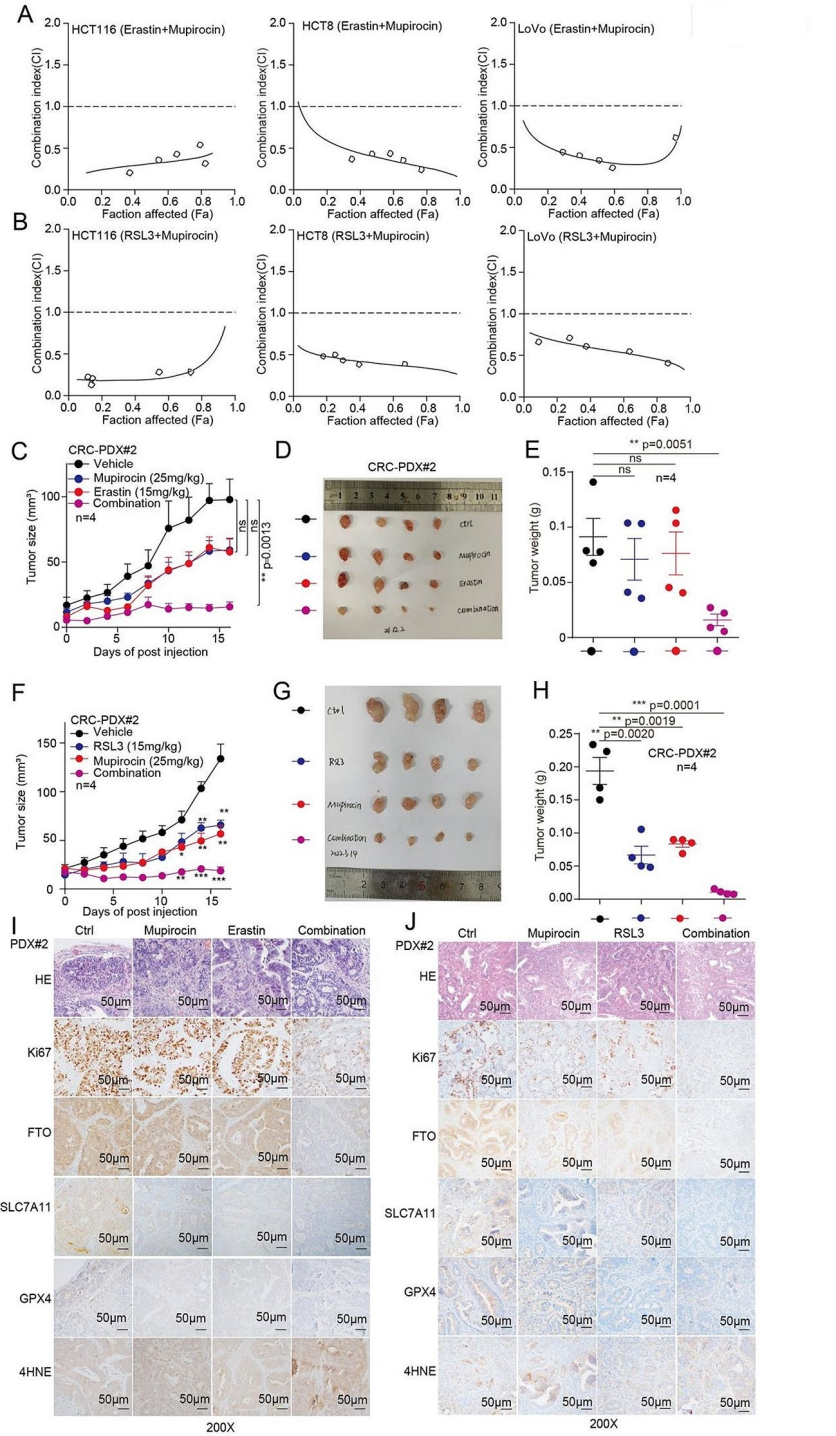
Mupirocin enhances the anti-tumor effects of Erastin and RSL3. (**A**-**B**) The coefficient of combination index (CI) value were calculating by cell number counting assay in CRC cells. (**C**) Tumor growth was compared between xenograft nude mice bearing with CRC PDX, which IP injection with Mupirocin alone, Erastin alone, and combination (*n* = 4). Tumor volume was calculated for each group at the indicated times. (**D**) All tumors from nude mouse were shown. (**E**) Tumor mass of xenograft nude mice injected with PDX tumor treated with treated with Mupirocin alone, Erastin alone, and combination (*n* = 4). (**F**) Tumor growth was compared between xenograft nude mice injected with PDX tumor treated with treated with Mupirocin alone, RSL3 alone, and combination (*n* = 4). (**G**) All tumors from nude mouse were shown. (**H**) Tumor mass of xenograft nude mice injected with PDX tumor treated with treated with Mupirocin alone, RSL3 alone, and combination (*n* = 4). (**I**-**J**) HE, Ki67, FTO, SLC7A11, GPX4, and 4HNE were analyzed by IHC in a representative PDX xenograft tumor treated with Mupirocin in the absence or presence of Erastin (or RSL3)(scale bar = 50 μm). (All error bars, mean values ± SEM, p values were determined by unpaired two-tailed Student’s t test of *n* = 3 independent biological experiments. *p < 0.05; **p < 0.01; ***p < 0.001)

Our findings demonstrated that Mupirocin regulates ferroptosis by targeting FTO, thus, we wonder whether targeting FTO by Mupirocin would also enhance the therapeutic efficacy of Erastin or RSL3, respectively. Firstly, we compared the sensitivity of CRC cells to Erastin or RSL3 treatment with or without Mupirocin and found that Mupirocin treatment increased the sensitivity of CRC cells to Erastin or RSL3 treatment based on cell number count and colony formation assay (Fig. S7a, d). Secondly, we further tested the synergistic effect of Mupirocin and Erastin or RSL3 by the Median-effect Method described by Chou and Talalay. Synergism (CI < 1) was observed between the two agents (Mupirocin and Erastin or RSL3) (Fig. 7a, b). While, the synergistic inhibition effect of Mupirocin and RSL3 (or Erastin) on cell proliferation were abolished in FTO knockdown group (Fig. S7e). These results suggested that Mupirocin and RSL3 (or Erastin) have synergistic inhibition on cell proliferation in a FTO-dependent manner.

To further evaluate its therapeutic effects of Mupirocin in combination with Erastin or RSL3, we established a PDX mouse model. As shown in Fig. 7c-h, the Mupirocin in combination with Erastin or RSL3 therapy significantly restrained tumor growth compared to the monotherapy and control group. There was no significant difference in body weight between the drug treatment group and the control group (Fig. S7f, g). Moreover, the PDX tumors IHC staining showed that Ki67 staining was reduced in the combination group (Fig. 7i, j). In addition, FTO, SLC7A11, and GPX4 expression were decreased, as well as the 4NHE levels were increased after Mupirocin and Erastin or RSL3 treatment (Fig. 7i, j). These implied that ferroptotic death occurred in the tumors of the combined drug group, resulting in an inhibition of tumor growth. Taken together, these results suggested that targeting FTO by Mupirocin enhances the anti-tumor effects of Erastin or RSL3 in CRC xenografts.

#### SLC7A11 and GPX4 expression is positively correlated with FTO in patients with colorectal cancer

Our previous study showed that FTO expression was upregulated in CRC tissues [18]. Here, in order to explore the correlation among FTO, SLC7A11, and GPX4 expression. We used another CRC tissues micro-array to determine the expression of FTO, SLC7A11, and GPX4 by IHC staining analysis. Our findings show that FTO, SLC7A11, and GPX4 protein are also highly expressed in patient tumor tissues than in normal tissues, respectively (Fig. S8a-f). What’s more, SLC7A11 and GPX4 expression positively correlated with FTO in CRC tissues (Fig. S8g, h). These data suggested that FTO, SLC7A11, and GPX4 expression are elevated in CRC tissues, and the expression of SLC7A11/GPX4 and FTO are positively correlated in CRC cancer.

## Discussion

FTO had been widely studied in various cancers and reported to play crucial roles in the regulation of tumorigenesis [16, 28–32]. However, the detail role and mechanism of FTO in promoting CRC tumorigenesis remains largely unknown. In this study, we demonstrated a significant oncogenic role played by FTO in promoting CRC tumorigenesis by protecting CRC from ferroptotic cell death through triggering both GPX4 and SLC7A11 expression, which guard CRC from ferroptotic damage by eliminating the accumulation of lipid peroxidation. Furthermore, through a series of screening and validation assays, we identified Mupirocin as a novel inhibitor of FTO, which showed synergistic effect of Mupirocin and Erastin or RSL3 on inhibiting the tumorigensis of CRC, which provides proof-of-concept evidence indicating the therapeutic potential of pharmacological targeting FTO plus with Erastin and RSL3 for treating CRC (Fig. S8i).

Ferroptosis is a newly discovered cell death with iron-dependent lipid peroxidation, which is induced by metabolic stress such as GSH depletion. The system Xc ^−^ is a heterodimer composed of two subunits, SLC7A11 and SLC3A2 [33], which mediate uptake of extracellular cystine and then convert it to cysteine in the cytoplasm. Cysteine was subsequently used to synthesize GSH, which was converted to GSSG by GPX4 and prevents lipid peroxide accumulation. Meanwhile, many studies suggest that Erastin (SLC7A11 inhibitor) or RSL3 (GPX4 inhibitor) could inhibit tumor progression and also increasing the chemotherapeutic effect of some chemotherapeutics [34]. However, whether ferroptosis inducer could inhibit CRC growth mediated by inducing ferroptosis remains unclear. In this study, we found that ferroptosis inducers significantly inhibited CRC cell viability mediated by inducing ferroptosis.

Evidencing studies show that the m^6^A modification was regulated by several of stimulation. For example, m^6^A modification is rapidly and transiently induced at DNA damage sites in response to UV [35]. ROS significantly induces global mRNA m^6^A levels by modulating ALKBH5 [36]. Shen et al. reported that ferroptosis inducing compounds increase the total levels of m^6^A modification in hepatic stellate cells [19]. However, the physiological signals that determine the m^6^A methylation status have not been fully explored. Here, we reported that the total levels of m^6^A modification are evidently increased upon exposure to ferroptosis-inducer (Erastin or RSL3) in CRC. Consistent with previous studies show that m^6^A modification was evidently increased in hepatic stellate cells by treated with ferroptosis-inducing compounds [19]. In addition, we identified that FTO was the mainly regulator, which is responsible for the m^6^A modification changed under ferroptosis inducer treatment. While, our results are different from Shen et al. study, they found that ferroptosis inducer treatment regulates m^6^A modification by increasing the expression of methylase METTL3 and decreasing the expression of FTO [19]. Although we found that the FTO protein level was decreased in CRC cells with ferroptosis inducer treatment, we did not explore the mechanism of FTO downregulation. Further studies are warranted to decipher the mechanism underlying the downregulation of FTO protein level when CRC cells exposure to ferroptosis inducer.

Evidencing studies showed the pharmacological system Xc ^−^ or GPX4 inhibition can lead to the accumulation of lipid peroxides, and then induce cell death in a ferroptosis dependent manner [37]. Herein, we also explored the underling mechanism which FTO inhibition on ferroptotic cell death. Mechanistically, on the one way, FTO reduces m^6^A methylation levels of SLC7A11/GPX4 transcripts, leading to increasing SLC7A11/GPX4 expression, in a m^6^A-YTHDF2-dependent manner. Thereby, FTO trigger both GPX4 and SLC7A11 expression to guard CRC from ferroptotic damage by eliminating the accumulation of lipid peroxidation in CRC. Thus, targeting the FTO-SLC7A11-GPX4 axis can induce ferroptotic cell death to therapy CRC. Here, we found that targeting FTO would enhance the chemotherapeutic effect of Erastin or RSL3. Taken together, these findings expand our understanding of the role and mechanisms of FTO, which make CRC cell able to progression under various types of adverse environmental changes, including cell death stimuli.

As given the role of FTO in protecting CRC from ferroptotic cell death. Thus, we want to explore the possibility by targeting FTO to kill the cancer. Interestingly, we develop Mupirocin as a novel inhibitor of FTO and Mupirocin induces CRC ferroptosis and inhibits tumor growth, as well as enhances the anti-tumor effects of Erastin or RSL3. Taken together, these findings suggest that targeting FTO by Mupirocin is a promising strategy for CRC therapy and also expands the chemotherapeutic effect of Erastin and RSL3.

## Conclusion

In summary, our results demonstrated that targeting FTO could significantly suppress cancer cell growth in a ferroptosis dependent manner. Mechanistically, we revealed that FTO protects CRC from ferroptotic cell death, not only by increasing the expression of SLC7A11, but also increasing antioxidant capacity, accumulation of lipid ROS, and ultimately the occurrence of oxidative damage and ferroptosis by enhancing the GPX4 activity. These findings shed light on new molecular mechanisms of CRC development and treatment regulated by m^6^A modification mediated ferroptosis and provide new insights into developing effective therapeutic strategies for treating CRC.

### Electronic supplementary material

Below is the link to the electronic supplementary material.

Supplementary Material 1

## Data Availability

All unique/stable reagents generated in this study are available from the Lead Contact with a completed Materials Transfer Agreement.

## Abbreviations

CRC: colorectal cancer
FTO: fat mass and obesity-associated protein
SLC7A11: solute carrier family 7 member 11
GPX4: glutathione peroxidase 4
MDA: malondialdehyde
m^6^A: N6-methyladenosine
RIP: RNA immunoprecipitation
DFO: deferoxamine
Fer-1: ferrostatin1
