# Correction: Targeting FTO induces colorectal cancer ferroptotic cell death by decreasing SLC7A11/ GPX4 expression

**DOI:** 10.1186/s13046-024-03051-6

**Published:** 2024-05-02

**Authors:** Yaya Qiao, Meng Su, Huifang Zhao, Huanle Liu, Chenxi Wang, Xintong Dai, Lingling Liu, Guangju Liu, Huanran Sun, Mingming Sun, Jiyan Wang, Zhen Li, Jun Fan, Quan Zhang, Chunshen Li, Fangmin Situ, Jun Xue, Zhenghu Jia, Chunze Zhang, Shuai Zhang, Changliang Shan

**Affiliations:** 1 State Key Laboratory of Medicinal Chemical Biology, College of Pharmacy and Tianjin Key Laboratory of Molecular Drug Research, Nankai University, Tianjin, 300350 China; 2 School of Life Science and Bio-Pharmaceutics, Shenyang Pharmaceutical University, LiaoningShenyang, 117004 China; 3 School of Integrative Medicine, Tianjin University of Traditional Chinese Medicine, Tianjin, 301617 China; 4 Guangzhou Key Laboratory for Clinical Rapid Diagnosis and Early Warning of Infectious Diseases, KingMed School of Laboratory Medicine, Guangzhou Medical University, GuangdongGuangzhou, 510180 China; 5 Department of Medical Biochemistry and Molecular Biology, School of Medicine, Guangdong Second Provincial General Hospital, Jinan University, Guangzhou, 510632 China; 6 College of Chinese and Culture, Jinan University, Guangzhou, 510632 China; 7 Department of General Surgery, The First Affiliated Hospital of Hebei North University, Zhangjiakou, 075000 China; 8 The First Affiliated Hospital, Biomedical Translational Research Institute and Guangdong Province Key Laboratory of Molecular Immunology and Antibody Engineering, Jinan University, Guangzhou, 510632 China; 9 Tianjin Key Laboratory for Modern Drug Delivery & High-Efficiency, Collaborative Innovation Center of Chemical Science and Engineering, School of Pharmaceutical Science and Technology, Tianjin University, Tianjin, 300193 China; 10 Department of Colorectal Surgery, Tianjin Union Medical Center, Nankai University, Tianjin, 300121 China

**Correction** **:** **J Exp Clin Cancer Res 43, 108 (2024)**

**https://doi.org/10.1186/s13046-024-03032-9**

Following publication of the original article [1], authors identified errors in the published Supplementary Materials. They were overlooked and incorrect files were mistakenly processed during production.

The correction does not affect the overall result or conclusion of the article. The original article [1] has been corrected.

### Supplementary Information

**Supplementary Material 1.**
