# Supplementary material for: Targeting FTO induces colorectal cancer ferroptotic cell death by decreasing SLC7A11/GPX4 expression
Source: J Exp Clin Cancer Res. 2024 Apr 10;43:108. doi: 10.1186/s13046-024-03032-9 (PMC11005233; doi:10.1186/s13046-024-03032-9)

**Materials and Methods**

**CONTACT FOR REAGENT AND RESOURCE SHARING**

**Lead contact**

Further information and requests for resources and reagents should be directed to and will be fulfilled by the lead contact, Changliang Shan ([changliangshan@nankai.edu.cn](mailto:changliangshan@nankai.edu.cn)).

**Materials availability**

All unique/stable reagents generated in this study are available from the Lead Contact with a completed Materials Transfer Agreement.

**Data and code availability**

All data are provided in the manuscript and supplement. The sequence and sample data have been deposited in NCBI database under Sequence Read Archive (SRA) (Bioproject identification number: PRJNA907157, PRJNA909448, and PRJNA909576), which includes our RNA-sequencing (RNA-seq) and MeRIP-seqencing (m6A-seq).

**Cell culture**

The colorectal cancer (CRC) cells LoVo, HCT116, and HCT8 were cultured in RPMI1640 (Thermo Fisher Scientific, MA, USA) supplemented with 10% fetal bovine serum (FBS, ExCell Bio, Shanghai, China). HEK 293T was cultured in Dulbecco’s modified Eagle’s medium (DMEM, Thermo Fisher Scientific, MA, USA) supplemented with 10% FBS, antibiotics, and 10mM HEPES, and used for lenti-virus package. Cell lines were purchased from the were purchased from the American Type Culture Collection (ATCC, USA). The cell culture assay were performed with [mycoplasma](https://www.sciencedirect.com/topics/pharmacology-toxicology-and-pharmaceutical-science/mycoplasma)-free cells, all the cell lines have been authenticated using short tandem repeat (STR) profiling within the last three years, and none of the cells used in the experiments were more than thirty generations.

**Western blot**

The cells were harvested and lysed with the RIPA lysis buffer (150 mM NaCl, 10 mM HEPES Buffer Solution, 1 % TritonX-100, 5 mM Naprophosphate, 5 mM NaF, 2 mM Na3VO4, 20 mM Glycerol phosphate) at 4°C. The cell lysate was centrifuged and the protein concentration was quantified by Ultra Trace Ultraviolet Spectrophotometer. An equal amount of cell lysate from each sample was loaded to the SDS-PAGE. The proteins were transferred onto PVDF membranes (Millipore, CA, USA), blocked with 5% skim milk and incubated with antibodies against FTO, SLC7A11, GPX4, YTHDF2, and β-actin etc, respectively. HRP conjugated Goat Anti-mouse or Anti-rabbit IgG was used as secondary antibody. The signal was detected by Immobilon Western HRP Kit (Millipore, CA, USA).

**RNA-sequencing (RNA-seq)**

HCT8-shCtrl and HCT8-shFTO#3 cells (or HCT8-DMSO and HCT8-Mupirocin) were collected and resuspended with TRIzol, and RNA-seq was performed using a sequencing platform (Lianchuan Biotechnology, Hangzhou, China). The differentially expressed genes were further analyzed by Gene Ontology (GO) and Gene Set Enrichment Analysis (GSEA).

**Patient samples**

CRC tissue microarrays (TableS1-S3 n = 44) were provided by Tianjin Union Medical Center (Tianjin, China). These microarrays included 44 colorectal cancer tissues, and 44 normal colorectal tissues. The relevant characteristics of queue are shown in Tables S1-S3. Fresh CRC tumor tissue used to establish the patient-derived tumor xenograft (PDX) or patient-derived organoid (PDO) were provided by Tianjin Union Medical Center (Tianjin, China). The research protocol was approved by the Ethics Committee of the Institute of Nankai University and Tianjin Union Medical Center.

**Evaluation of IHC staining**

All specimens were examined by investigator (Y Qiao) who did not possess knowledge of the clinical data. Briefly, the IHC staining for FTO or SLC7A11 or GPX4 was semi-quantitatively scored as ‘-’ (negative, no or less than 5% positive cells), ‘+’ (5-25% positive cells), and ‘++’ (more than 25% positive cells, considered as strongly positive).

**Animal model of Patient-Derived tumor Xenograft (PDX)**

The BALB/c nude mice were obtained from Beijing Vital River Laboratory Animals Technology Co. Ltd, and were bred and subjected to the xeno-transplantation model. For PDX model, the fresh CRC tumor tissues used to establish the PDX mice model were provided from Tianjin Union Medical Center (Tianjin, China). The patient tumors were divided into approximately 1 mm^3^ using sterile surgical scissors and then inoculated on flank of nude mice using a mouse tumor inoculator. After the tumor grows to a suitable size, the tumor was divided into approximately 1 mm^3^ and then subcutaneously inoculated on flank of another mice. For knockdown FTO mice model, when tumor volumes reached approximately 20 mm^3^, mice were randomly distributed into group. To investigate the effect of FTO on tumori genicity of cancer, shFTO or shCtrl virus were injected precisely into the center of the xenografted tumors first three days for three times. For medication mice model, intraperitoneal injection of drug was started one week after inoculation. Tumor growth was recorded by measurement of two perpendicular diameters using the formula (tumor volume =4π/3*(length/2)*(width/2)^2^). All experimentation followed protocol approved by Nankai University Animal Care and Use Committee.

**Establishment of patient-derived organoid (PDO)**

Primary tumor tissues obtained during surgery were cleaned with Washing solution (Precedo, Hefei, China) and cut into small pieces of 1-3mm^3^. Digestive Enzyme (Precedo, Hefei, China) is then used to digest the tissue at 37 ° C to the extent of 3-10 cell clumps. Digested cell clumps were collected and then re-suspended using Intestine Carcinoma Organoid Medium (Precedo, Hefei, China) and mixed by adding reduced growth factor basement membrane matrix Type2，(BME，R&D Systems) at a final volume of 70%. The 24-well plates were placed in a cell incubator at 37℃ to solidified the BME completely. Each well was added with 500 μL complete medium and placed in a cell incubator with 5% CO_2_ at 37℃ for further culture.

**Drug treatment of organoids**

Primary tumor cells were implanted into 96-well and 48-well cell culture plates, and organoids of 50-100 μm were formed after 5-7 days. Mupirocin of different concentrations was added into the organoid medium with treating for 5 days. Organoid cell viability was determined by CellTiter-Glo 3D reagent (G9683, Promega, MI, USA) under different treatment conditions, and morphological changes of organoids were recorded by electron microscope.

**m6A Dot blot Assay**

Dot blot assay was performed to determine the global m^6^A abundance of total RNA. In brief, total RNA was mixed with SSC buffer and denatured at 65°C for 5 min. Then, the RNA samples were loaded on the Amersham Hybond-N+ membrane (RPN119B, GE Healthcare), and crosslinked to the membrane by UV. The membrane was stained by methylene blue for control. Then, the membrane was blocked with 5% skim milk and incubated with m6A antibody overnight at 4°C. After rinsed with PBST, the membrane was incubated with secondary antibody for 1 h. The signal was detected by Immobilon Western HRP Kit (Millipore, USA).

**Measurement of GSH and GSSG**

Intracellular GSH and GSSG levels were assayed using GSH/GSSG assay kit (Beyotime, Shanghai, China) according to the manufacturer’s instructions. Briefly, quantified cells were washed with cold PBS and pelleted. Add protein removal reagent to the cell pellet and vortex well. The samples were then rapidly freeze-thawed twice using liquid nitrogen and a 37°C water bath, after which the samples were placed on ice for five minutes and centrifuged at 10 000g for 10 minutes at 4℃. Take the supernatant for subsequent Total Glutathione and GSSG assays. Total Glutathione and GSSG were measured in the supernatant by reading the fluorescence at 405 nm. Finally, according to the measured total glutathione content and GSSG content, the GSH content can be calculated (GSH=Total Glutathione-GSSG*2).

**Measurement of MDA**

Intracellular MDA levels were assayed using MDA assay kit (Beyotime, Shanghai, China) according to the manufacturer’s instructions. Briefly, the cells were harvested and lysed with the RIPA lysis buffer at 4°C. The cell lysate was centrifuged and the protein concentration was quantified by Ultra Trace Ultraviolet Spectrophotometer. Take the quantified supernatant, add MDA detection working solution, heat in water bath for 15minutes, cool to room temperature, centrifuge at 1000g for 10minutes, and then measure the absorbance at 532nm. Calculate MDA content based on the determined protein sample concentration.

**Real-Time Quantitative PCR (RT-qPCR)**

Total RNA was isolated using TRIzol reagent (Thermo Fisher Scientific, MA, USA), followed by reverse transcription using PrimeScript RT kit with gDNA Eraser (RR047A, Takara, Dalian, China). Finally, RT-qPCR reactions were performed with TB Green™ Premix Ex Taq™ II (RR820A, Takara, Dalian, China) on a CFX96™ real-time PCR detection system (Bio-Rad, CA, USA) and primers listed in the KEY RESOURCES TABLE. Gene expression was calculated using the comparative ΔΔCT method with the actin for normalization.

**Immunohistochemistry**

Immunohistochemistry was performed on paraffin-embedded sections. Deparaffinizing and rehydrating tissue sections using standard protocols. Antigen retrieval was performed by boiling the samples in citrate buffer for 40 minutes after deparaffinization of tissue sections. Use of 3% catalase inhibits endogenous peroxidase activity. Sections were blocked in 10% NGS in PBS or PBST and incubated overnight at 4°C in primary antibody. Sections were rinsed in PBS or PBST and visualized with DAB before counterstaining with hematoxylin. Staining was quantified using Image J software.

**Measurement of m6A level by enzyme linked immunosorbent assay (ELISA)**

m6A levels were assayed using m6A assay kit according to the manufacturer’s instructions (Epigentek P-9005). Briefly, cells were collected and total RNA was extracted, 200ng total RNA was taken from each reaction for determination. The Binding Solution was added to the wells and RNA, 37℃ for 1h to bind RNA, and then the reaction solution was discarded and washed three times with Washing buffer. Then the Captured Antibody was added and reacted at room temperature for 1h. After discarding the solution, the wells were washed again for three times. Add the Detection antibody and react at room temperature for 30 minutes, then discard and wash the wells four times. Add the Enhancer Solution and react at room temperature for 30 minutes before discarding, and wash the wells five times. After Developer Solution was added for 1-10min, Stopping Solution was added to terminate the reaction and transferred to 96-well plate to detect its light absorption value at 450nM.

**Combination Index (CI)**

The CRC cells were seeded into 24-well plates, and the appropriate concentration gradients of A/B inhibitors were added on the second day of seeding. The cells were counted -after 72 hours and the FA/CI value was analyzed using CONPUSY software.

**Purification of prokaryotic protein**

Prokaryotic protein expression bacteria were collected and resuspended with lytic buffer. The bacterial solution was broken by ultrasound, and the supernatant of the broken bacterial solution was centrifuged and added to the adsorption column of His-tag. Different concentrations of imidazole were used to wash the impurity protein, and the target protein was eluted with high concentration of imidazole. The eluted target protein was concentrated by ultrafiltration tube.

**The cellular thermal shift assay (CETSA) in cell-free system**

The cells transfected with FTO (WT or mutant) were harvested and lysed with the RIPA lysis buffer at 4°C. The cells lysate was divided into two parts, one of which was added with the appropriate concentration of the drug, and the other was added with an equal volume of DMSO. Incubating the cell lysate for 25 min at 25°C. Afterwards, the cell lysates of each group were equally divided into PCR tubes, and then heat-shocked with different concentration gradients for 30 minutes. The signal was detected by Immobilon Western HRP Kit (Millipore, USA).

**Luciferase reporter gene assay**

We constructed GPX4 mRNA CDS wild-type (WT) containing the site2 m6A motif-sequence into the pmirGLO (7,350 bp) dual luciferase reporter vector (Promega, Madison, WI, USA)，GPX4 mRNA CDS were digest from pCMV-Flag-GPX4 with Xho I and  NheI. A mutated segment of the GPX4 mRNA CDS site2 (MUT), which the m6A motif-sequence GGAC was converted to GGCC were constructed in pmirGLO using the Fast Mutagenesis System (TRAN, Beijing, China). When the cells density reaches 70% confluence after seeded in 6-well plates, then we co-transfected with FLAG-YTHDF2 with pmirGLO-GPX4 WT or MUT plasmids using PEI (Sigma-Aldrich). Cells were lysed and assayed for luciferase activity 48h after transfection. Protein extracts were analyzed in a luminometer. Luciferase reports assay was performed using the Dual-Luciferase Reporter assay System (Beyotime) according to the manufactureeer`s instructions.

**Synthesis of Mupirocin probe**

The solution of Mupirocin (21.9 mg, 0.44 mmol), EDCI (83.7 mg, 0.44 mmol), HOBt (54.0 mg, 0.40 mmol) and CH_2_Cl_2_ (5 mL) was stirred for 0.5 h at room temperature, and then 2-(3-(but-3-yn-1-yl)-3H-diazirin-3-yl)ethan-1-amine (50 mg, 0.36 mmol) was added. The resulting mixture was stirred overnight at room temperature. The reaction mixture was washed with saturated NaHCO_3_ and saturated NaCl. The organic phase was dried over anhydrous MgSO_4_ and concentrated under reduced pressure to give the crude product, which was purified by silica gel column chromatography to afford Mupirocin probe (104.4 mg, 46%). ^1^H NMR (400 MHz, CDCl_3_) δ 5.78 – 5.71 (m, 2H), 5.29 (s, 1H), 4.05 (t, *J* = 6.6 Hz, 2H), 3.93 – 3.71 (m, 4H), 3.57 – 3.40 (m, 2H), 3.08 (q, *J* = 6.5 Hz, 2H), 2.79 (td, *J* = 5.8, 2.2 Hz, 1H), 2.70 (dd, *J* = 7.9, 2.2 Hz, 1H), 2.64 – 2.55 (m, 1H), 2.25 (dd, *J* = 14.8, 9.2 Hz, 1H), 2.20 – 2.13 (m, 5H), 2.06 – 1.95 (m, 5H), 1.74 – 1.56 (m, 9H), 1.38 – 1.28 (m, 9H), 1.27 (s, 1H), 1.24 (d, *J* = 2.3 Hz, 1H), 1.20 (d, *J* = 6.3 Hz, 3H), 0.92 (d, *J* = 7.0 Hz, 3H); ^13^C NMR (100 MHz, CDCl_3_) δ 173.49, 166.84, 156.86, 117.54, 82.72, 74.88, 71.37, 70.36, 69.45, 68.98, 65.37, 63.79, 61.33, 55.63, 53.45, 42.84, 39.55, 36.72, 34.30, 32.49, 32.15, 31.60, 29.69, 29.11, 28.98, 28.58, 26.85, 25.93, 25.60, 20.83, 19.12, 13.23, 12.75; HRMS (ESI) calcd for C_33_H_53_N_3_NaO_8_ [M+Na]^+^ 642.3725; found 642.3731.

**In situ pull-down assay (Probe pull‑down assay)**

In the in situ pull-down experiments, cells were inoculated into 10cm dish, and the Mupirocin probe was added with appropriate concentration 24h later after seeding, and incubated for 6h. Cell precipitates were then collected and cells were lysed with cell lysate. After centrifugation, the supernatant was collected and a mix of TBTA (0.1 mM), TCEP (1 mM), biotin-N3 (100 µM) and CuSO_4_ (1 mM). Then, excess pre-cooled methanol was added and incubated at −80°C for 30 minutes to precipitate the protein. After centrifugation (15000g, 15min), the precipitated protein was dissolved and incubated overnight with streptavidin magnetic beads (Thermo Fisher Scientific, MA, USA) at 4℃. Then, streptavidin beads were cleaned with washing buffer for 3 times, and bead-binding proteins were collected for detection by western blot.

**KEY RESOURCES TABLE**

| REAGENT or RESOURCE | SOURCE | IDENTIFIER |
| --- | --- | --- |
| Antibodies | | |
| Rabbit anti-FTO | Proteintech | Cat# 27226-1-AP, RRID:AB_2880809 |
| Rabbit anti-METTL3 | Proteintech | Cat# 15073-1-AP, RRID:AB_2142033 |
| Rabbit anti-METTL14 | Proteintech | Cat# 26158-1-AP, RRID:AB_2800447 |
| Rabbit anti- WTAP | Proteintech | Cat#10200-1-AP, RRID: AB_2216349 |
| Rabbit anti-SLC7A11 | Proteintech | Cat# 26864-1-AP, RRID:AB_2880661 |
| Rabbit anti-GPX4 | Abcam | Cat# ab125066, RRID: AB_10973901 |
| Rabbit anti-4HNE | Abcam | Cat# ab46545, RRID: AB_722490 |
| Rabbit anti-YTHDF2 | Proteintech | Cat# 24744-1-AP, RRID:AB_2687435 |
| Rabbit anti-FLAG | Proteintech | Cat#20543-1-AP, RRID:AB_11232216 |
| Rabbit anti-Ki67 | Proteintech | Cat# 27309-1-AP, RRID:AB_2756525 |
| Mouse anti-β-actin | Proteintech | Cat# 66009-1-Ig, RRID:AB_2687938 |
| Rabbit anti-m6A | Abcam | Cat# ab151230, RRID:AB_2753144 |
| Rabbit anti-m6A | Abcam | Cat# ab190886, |
| Biological Samples | | |
| CRC tissue microarrays (TableS1-S3)  Fresh CRC tumor tissue for PDX  Fresh CRC tumor tissue for PDO | Department of Colorectal Surgery, Tianjin Union Medical Center | N/A |
| Chemicals, peptides, and recombinant proteins | | |
| Rhein | Sangon Biotech | Cat# A506552 |
| Erastin | MedChemExpress | Cat# HY-15763 |
| RSL3 | MedChemExpress | Cat# HY-100218A |
| Ferrostatin-1 | MedChemExpress | Cat# HY-100579 |
| Deferoxamine | MedChemExpress | Cat# HY-B1625 |
| 3-Methyladenine | MedChemExpress | Cat# HY-19312 |
| Z-VAD-fmk | MedChemExpress | Cat# HY-16658B |
| Actinomycin D | MedChemExpress | Cat# HY-17559 |
| Luteolin 7-O-glucuronide | MedChemExpress | Cat# HY-N1463 |
| Vitexin-4''-O-glucoside | MedChemExpress | Cat# HY-N5073 |
| Rhodiosin | MedChemExpress | Cat# HY-N2425 |
| Lithospermic acid | MedChemExpress | Cat# HY-N0823 |
| Plantamajoside | MedChemExpress | Cat# HY-N0031 |
| Cosmosiin | MedChemExpress | Cat# HY-N0578 |
| Mupirocin | MedChemExpress | Cat# HY-B0958 |
| Mupirocin | Syhuanye | Cat# B25502 |
| Polybrene | Sigma | Cat# H9268 |
| Polyethylenimine (PEI) | Polysciences | Cat# 23966 |
| TRIzol | Thermo Fisher Scientific | Cat# 15596018 |
| Washing solution | Precedo | Cat# PRS-TCR-1 |
| Digestive enzyme | Precedo | Cat# PRS-TDE-2 |
| Intestine Carcinoma Organoid Medium | Precedo | Cat# PRS-ICM-3D |
| Reduced Growth Factor Basement Membrane Matrix ,Type 2 | R&D Systems | Cat# 3533-010-02P |
| Critical Commercial Assays | | |
| GSH and GSSG Assay Kit | Beyotime | Cat# S0053 |
| Lipid Peroxidation MDA Assay Kit | Beyotime | Cat# S0131M |
| PrimeScript RT reagent Kit with gDNA Eraser | TAKARA | Cat# RR047A |
| TB Green™ Premix Ex Taq™ II | TAKARA | Cat# RR820A |
| m6A Elisa kit | Epigentek | Cat# P-9005 |
| CellTiter-Glo 3D reagent | Promega | Cat# G9681 |
| Experimental models: cell lines | | |
| LoVo | This paper | N/A |
| HCT116 | This paper | N/A |
| HCT8 | This paper | N/A |
| HEK 293T | This paper | N/A |
| Oligonucleotides | | |
| FTO F | ACTTGGCTCCCTTATCTGACC | N/A |
| FTO R | TGTGCAGTGTGAGAAAGGCTT | N/A |
| SLC7A11 F | TCTCCAAAGGAGGTTACCTGC | N/A |
| SLC7A11 R | AGACTCCCCTCAGTAAAGTGAC | N/A |
| GPX4 F | GAGGCAAGACCGAAGTAAACTAC | N/A |
| GPX4 R | CCGAACTGGTTACACGGGAA | N/A |
| YTHDF2 F | CCTTAGGTGGAGCCATGATTG | N/A |
| YTHDF2 R | TCTGTGCTACCCAACTTCAGT | N/A |
| ALKBH5 F | CGGCGAAGGCTACACTTACG | N/A |
| ALKBH5 R | CCACCAGCTTTTGGATCACCA | N/A |
| WTAP F | CTTCCCAAGAAGGTTCGATTGA | N/A |
| WTAP R | TCAGACTCTCTTAGGCCAGTTAC | N/A |
| METTL3 F | TTGTCTCCAACCTTCCGTAGT | N/A |
| METTL3 R | CCAGATCAGAGAGGTGGTGTAG | N/A |
| METTL14 F | AGTGCCGACAGCATTGGTG | N/A |
| METTL14 R | GGAGCAGAGGTATCATAGGAAGC | N/A |
| SLC7A11 site1 F | CTGGCATTTGGACGCTACATTC | N/A |
| SLC7A11 site1 R | GCTCCAGCTGACACTCATG | N/A |
| SLC7A11 site2 F | GCAAGCTCACAGCAATTCTG | N/A |
| SLC7A11 site2 R | GTGGCAACCGCGTAATAC | N/A |
| SLC7A11 site3 F | CTGGAAGTTGTACCAGAAGAAG | N/A |
| SLC7A11 site3 R | CTCAAGAATTGTGCGACTCATAG | N/A |
| SLC7A11 site4 F | GGACTTGAGATCTTGGCAATC | N/A |
| SLC7A11 site4 R | CTCAAGAATTGTGCGACTCATAG | N/A |
| SLC7A11 site5 F | GAGTCGCACAATTCTTGAGTCTC | N/A |
| SLC7A11 site5 R | GCCGTGCTAACATATGTTGTAGAG | N/A |
| SLC7A11 site6 F | CCTGCCATTGTAATGCC | N/A |
| SLC7A11 site6 R | GGAACTGTGTCTTCTCATG | N/A |
| SLC7A11 site7 F | GGAAGATGGCTGACTTCGGTTC | N/A |
| SLC7A11 site7 R | CCGTCATGCATTGCTAACGTAAC | N/A |
| SLC7A11 site8 F | CATGAACTGTAATCAGTCTACAGG | N/A |
| SLC7A11 site8 R | CGAGATCCACCTATGCACAG | N/A |
| SLC7A11 site9 F | CCATGAATGTATGCTCATACTCG | N/A |
| SLC7A11 site9 R | CTCCAAGAAGAGGATCAGATTAC | N/A |
| SLC7A11 site10 F | GAATACAGACATTGGCTCCA | N/A |
| SLC7A11 site10 R | GCCTTAACACATATGATGCTC | N/A |
| SLC7A11 site11 F | CTGCATCCACATTCCAATC | N/A |
| SLC7A11 site11 R | CACATATCACATGCTTGTGC | N/A |
| GPX4 site1 F | TGCGCGCTCCATGCACGAGTTT | N/A |
| GPX4 site1 R | CACGTTGGTGACGATGCACACGAA | N/A |
| GPX4 site2 F | CAAGTGGAACTTCACCAAG | N/A |
| GPX4 site2 R | CACACACTTGTGGAGCT | N/A |
| GPX4 site3 F | CTGCCTGCAAACCTGCTGGT | N/A |
| GPX4 site3 R | CTGTTTATTCCCACAAGGTAG | N/A |
| GPX4 mut F | GAAATAGTGGGGCAGGGCCTTCTCTATCACCAG | N/A |
| GPX4 mut R | CTGGTGATAGAGAAGGCCCTGCCCCACTATTTC | N/A |
| shRNA | | |
| pLKO.1-puro | TranSheepBio | N/A |
| pLKO.1-puro-FTO shRNA | TranSheepBio | N/A |
| pLKO.1-puro-METTL3 shRNA | TranSheepBio | N/A |
| pLKO.1-puro-METTL14 shRNA | TranSheepBio | N/A |
| pLKO.1-puro-SLC7A11 shRNA | TranSheepBio | N/A |
| pLKO.1-puro-GPX4 shRNA | TranSheepBio | N/A |
| pLKO.1-puro-ALKBH5 shRNA | TranSheepBio | N/A |
| Recombinant DNA | | |
| pCDNA3.0-Flag-FTO | From Yang Caiguang | N/A |
| pCDNA3.0-Flag-FTO R96A | From Yang Caiguang | N/A |
| pCDNA3.0-Flag-FTO H231A/D233A | From Caiguang Yang | N/A |
| pCDNA3.0-SLC7A11 | This paper | N/A |
| pCMV-Flag-GPX4 | From Quan Zhang | N/A |
| pmirGLO | From Luyuan Li | N/A |
| pmirGLO-GPX4 CDS wt | This paper | N/A |
| pmirGLO-GPX4 CDS site 2 mut | This paper | N/A |
| Software | | |
| Prism-5.01  Prism-8.0.1 | GraphPad | N/A |
| Adobe Illustrator | Adobe | N/A |
| Image J-1.44p | National Institutes of Health | N/A |
| Leica Application Suite X -2.0.1 | Leica Microsystems | N/A |
| Compusyn | Chou TC.2010 | N/A |
| GSEA-4.0.3 | N/A | http://www.gsea-msigdb.org/gsea/index.jsp |
| igv-2.8.9 | N/A | http://software.broadinstitute.org/software/igv/ |

**Supplementary Tables**

**Table S1. Expression of FTO protein in colorectal cancer (CRC)**

| **Diagnosis** | **No.of case** | **FTO** | | | | **Positive cases rate (%)** | **strong positive cases rate(%)** |
| --- | --- | --- | --- | --- | --- | --- | --- |
|  |  | **-** | **+** | **++** | **+++** |  |  |
| CRC | 44 | 6 | 20 | 18 | 0 | 86.4%*** | 40.9%*** |
| Adjacent normal colon | 44 | 22 | 20 | 2 | 0 | 50.0% | 4.5% |

**Positive rate:** percentage of positive cases with + and ++ staining score.

**Strongly positive rate (high-level expression):** percentage of positive cases with ++ staining score.

*** P<0.001 compared with normal colon.

**Table S2. Expression of SLC7A11 protein in colorectal cancer (CRC)**

| **Diagnosis** | **No.of case** | **SLC7A11** | | | | **Positive cases rate (%)** | **strong positive cases rate(%)** |
| --- | --- | --- | --- | --- | --- | --- | --- |
|  |  | **-** | **+** | **++** | **+++** |  |  |
| CRC | 44 | 1 | 20 | 23 | 0 | 97.7%*** | **52.3%***** |
| Adjacent normal colon | 44 | 18 | 22 | 4 | 0 | 59.1% | 9.1% |

**Positive rate**: percentage of positive cases with + and ++ staining score.

**Strongly positive rate (high-level expression)**: percentage of positive cases with ++ staining score.

*** P<0.001 compared with normal colon.

**Table S3. Expression of GPX4 protein in colorectal cancer (CRC)**

| **Diagnosis** | **No.of case** | **GPX4** | | | | **Positive cases rate (%)** | **strong positive cases rate(%)** |
| --- | --- | --- | --- | --- | --- | --- | --- |
|  |  | **-** | **+** | **++** | **+++** |  |  |
| CRC | 44 | 4 | 23 | 17 | 0 | 90.1%*** | **38.6%***** |
| Adjacent normal colon | 44 | 17 | 24 | 3 | 0 | 61.4% | 6.8% |

**Positive rate**: percentage of positive cases with + and ++ staining score.

**Strongly positive rate (high-level expression)**: percentage of positive cases with ++ staining score.

*** P<0.001 compared with normal colon.

**Supplementary information for Synthesis of Mupirocin probe**

The route for synthesis of Mupirocin probe

^1^H NMR, ^13^C NMR, and HRMS copies of Mupirocin probe.

(1) ^1^H NMR

(2) ^13^C NMR

(3) HRMS


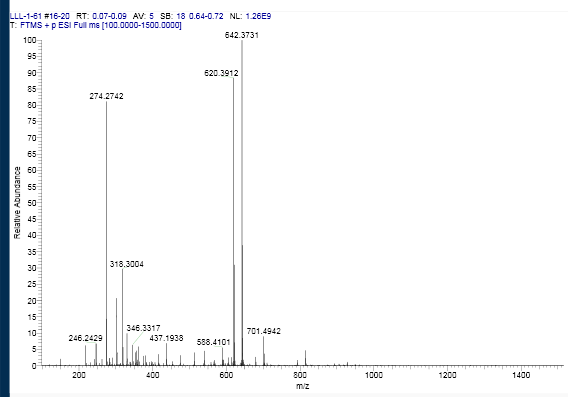


**Supplemental Figure legends**

**Figure S1 m6A modification is increased during ferroptosis cell death in CRC**

1. HCT116, HCT8, and LoVo cells treated with indicated doses of Erastin, and then harvested cells for counting cell number at indicated day 3.The IC50 of Erastin in CRC cells was determined by cell number counting assay.

(B) HCT116, HCT8, and LoVo cells treated with indicated doses of RSL3, and then harvested cells for counting cell number at indicated day 3. The IC50 of RSL3 in CRC cells was determined by cell number counting assay.

(C) HCT116, HCT8, and LoVo cells treated with indicated doses of Erastin, and then the cell proliferation was determined by cell colony formation assay.

(D) HCT116, HCT8, and LoVo cells treated with indicated doses of RSL3, and then the cell proliferation was determined by cell colony formation assay.

(E) HCT116, HCT8, and LoVo cells treated with 5 μM, 10 μM, and 20 μM of Erastin for 72h, and then the total RNA was harvested for dot blotting assay to determine the m6A levels.

(F) HCT116, HCT8, and LoVo cells treated with 200 nM, 400 nM, and 800 nM of RSL3 for 72h, and then the total RNA were harvested for dot blotting assay to determine the m6A levels.

(G) HCT116 and HCT8 cells pr-treated with 20 μM of Erastin (or 800 nM of RSL3) for 4h, subsequently treated with or without of DFO (100 nM) for 72h, and then the total RNA was harvested for dot blotting assay to determine the m6A levels.

(H) HCT116 and HCT8 cells pr-treated with 20 μM of Erastin (or 800 nM of RSL3) for 4h, subsequently treated with or without of Fer-1 (100 nM) for 72h, and then the total RNA was harvested for dot blotting assay to determine the m6A levels.

(All error bars, mean values ± SEM, p values were determined by unpaired two-tailed Student’s t test of n = 3 independent biological experiments. *p < 0.05; **p < 0.01; ***p < 0.001).

**Figure S2 FTO mediates m6A modification upregulation during ferroptosis and regulates ferroptosis**

1. HCT116 and HCT8 cells treated with 5 μM, 10 μM, and 20 μM of Erastin for 72h, and then the total RNA were harvested for qRT-PCR to examine the expression of FTO, ALKBH5, METTL3, METTL14, WTAP, and YTHDF2.
2. HCT116 and HCT8 cells treated with 200 nM, 400 nM, and 800 nM of RSL3 for 72h, and then the total RNA were harvested for qRT-PCR to examine the expression of FTO, ALKBH5, METTL3, METTL14, WTAP, and YTHDF2.
3. HCT116 and HCT8 cells treated with 5 μM, 10 μM, and 20 μM of Erastin for 72h. The lysates were collected for western blotting to examine the expression of WTAP.
4. HCT116 and HCT8 cells treated with 200 nM, 400 nM, and 800 nM of RSL3 for 72h. The lysates were collected for western blotting to examine the expression of WTAP.
5. HCT116 and HCT8 cells pr-treated with 20 μM of Erastin for 4h, subsequently treated with or without of Fer-1 (100 nM) for 72h. The lysates were collected for western blotting to examine the expression of FTO, ALKBH5, METTL3, WTAP, and METTL14.
6. HCT116 and HCT8 cells pre-treated with 800 nM of RSL3 for 4h, subsequently treated with or without of Fer-1 (100 nM) for 72h. The lysates were collected for western blotting to examine the expression of FTO, ALKBH5, METTL3, WTAP, and METTL14.
7. HCT116 and HCT8 cells pre-treated with 20 μM of Erastin for 4h, subsequently treated with or without of DFO (100 nM) for 72h. The lysates were collected for western blotting to examine the expression of WTAP.
8. HCT116 and HCT8 cells pre-treated with 800 nM of RSL3 for 4h, subsequently treated with or without of DFO (100 nM) for 72h. The lysates were collected for western blotting to examine the expression of WTAP.
9. FTO knockdown or vector control HCT8 and HCT116 cells treated with 20 μM of Erastin for 72h, and then the total RNA was harvested for dot blotting assay to determine the m6A levels.
10. FTO knockdown or vector control HCT8 and HCT116 cells treated with 800 nM of RSL3 for 72h, and then the total RNA was harvested for dot blotting assay to determine the m6A levels.
11. ALKBH5 knockdown or vector control HCT116 and HCT8 cells treated with 20 μM of Erastin for 72h, and then the total RNA was harvested for dot blotting assay to determine the m6A levels.
12. ALKBH5 knockdown or vector control HCT116 and HCT8 cells treated with 800 nM of RSL3 for 72h, and then the total RNA was harvested for dot blotting assay to determine the m6A levels.
13. The lysates were collected from FTO knockdown or vector control CRC cells for western blotting to examine the expression of FTO, ALKBH5, METTL3, and METTL14.
14. The malondialdehyde (MDA) concentration was detected using MDA assay kits in Rhein treated or vector control CRC cells.
15. The GSH/GSSG ratio was detected using GSH/GSSG assay kits in Rhein treated or vector control CRC cells.
16. The malondialdehyde (MDA) concentration were detected using MDA assay kits in FTO knockdown or vector control HCT116 and HCT8 cells exogenous expression with or without a shRNA-resistant FTO.
17. The GSH/GSSG ratio was detected using GSH/GSSG assay kits in FTO knockdown or vector control HCT116 and HCT8 cells exogenous expression with or without a shRNA-resistant FTO.
18. The GSH/GSSG ratio was detected using GSH/GSSG assay kits in FTO knockdown or vector control CRC cells with or without GSH treatment.

(All error bars, mean values ± SEM, p values were determined by unpaired two-tailed Student’s t test of n = 3 independent biological experiments. *p < 0.05; **p < 0.01; ***p < 0.001).

**Figure S3 Targeting FTO enhances the anti-tumor effects of Erastin and RSL3.**

(A) Cell survival was determined by colony formation assay in CRC cells treated with Rehin alone, Erastin alone, and combination.

(B) Cell survival was determined by colony formation assay in CRC cells treated with Rehin alone, RSL3 alone, and combination.

(C) Cell proliferation was determined in HCT116, HCT8 and LoVo cells with stable FTO knockdown when treated with or without 100 nM of DFO, and then harvested cells for counting cell number at indicated day 1, day 2, day 3, and day 4.

(D) Cell survival was determined in HCT116 and HCT8 cells with stable METTL3 or METTL14 knockdown when treated with 0 μM, 4 μM, 8 μM, and 15 μM of Erastin, and then harvested cells for counting cell number at indicated day 3.

(E) Cell survival was determined in HCT116 and HCT8 cells with stable METTL3 or METTL14 knockdown when treated with 0 nM, 100 nM, 200 nMand 400 nM of RSL3, and then harvested cells for counting cell number at indicated day 3.

(F) Cell survival was determined in HCT116 and HCT8 cells with stable ALKBH5 knockdown when treated with 0 μM, 4 μM, 8 μM, and 15 μM of Erastin, and then harvested cells for counting cell number at indicated day 3.

(G) Cell survival was determined inin HCT116 and HCT8 cells with stable ALKBH5 knockdown when treated with 0 nM, 100 nM, 200 nM and 400 nM of RSL3, and then harvested cells for counting cell number at indicated day 3.

(H) Tumor growth was compared between xenograft nude mice bearing with CRC PDX injected with FTO shRNA virus and control shRNA.

(I) All tumors from nude mouse are shown.

(J) Tumor mass in xenograft nude mice bearing with CRC PDX injected with FTO shRNA virus and control shRNA virus.

(K) HE, Ki67, FTO, SLC7A11, GPX4, and 4HNE were analyzed in a representative PDX xenograft tumor by IHC (scale bar = 50 μm).

(All error bars, mean values ± SEM, p values were determined by unpaired two-tailed Student’s t test of n = 3 independent biological experiments. *p < 0.05; **p < 0.01; ***p < 0.001).

**Figure S4 FTO enhances the expression of SLC7A11 and GPX4 in CRC cells.**

(A) Volcano plot showing genome-wide mRNA expression in FTO knockdown or vector control HCT8 cells.

(B) Heatmap indicated the mRNA expression level of ferroptosis-related genes in FTO knockdown or vector control HCT8 cells.

(C)The relative abundance of m6A sites along GPX4 mRNA in FTO knockdown cells and control cells, as detected by m6A-seq.

(D) The m6A modification levels on SLC7A11 in CRC cells were examined by the MeRIP-qPCR.

(E) The m6A modification levels on GPX4 in CRC cells were examined by the MeRIP-qPCR..

(F) RIP-qPCR analysis binding between the reader protein and SLC7A11 mRNA.

(G) RIP-qPCR analysis binding between the reader protein and GPX4 mRNA.

1. HCT116 and HCT8 cells treated with 20 μM of Erastin (or 800 nM of RSL3) (4h) in the absence or presence of DFO (100 nM) for 72h. The lysates were collected for western blotting to examine the expression of SLC7A11, and GPX4.
2. HCT116 and HCT8 cells treated with 20 μM of Erastin (or 800 nM of RSL3)(4h) in the absence or presence of Fer-1 (100 nM) for 72h. The lysates were collected for western blotting to examine the expression of SLC7A11, and GPX4.
3. The relative luciferase activity of GPX4 CDS WT and mut in the absence or presence of Flag-YTHDF2.

(All error bars, mean values ± SEM, p values were determined by unpaired two-tailed Student’s t test of n = 3 independent biological experiments. *p < 0.05; **p < 0.01; ***p < 0.001).

**Figure S5 FTO regulates ferroptosis and cell proliferation via SLC7A11/GPX4.**

(A) The expression of SLC7A11 and GPX4 were examined by western blotting in FTO knockdown or vector control CRC cells with or without exogenous expression of SLC7A11.

(B) The expression of GPX4 was examined by western blotting in FTO knockdown or vector control CRC cells with or without exogenous expression of GPX4.

(C) The cell proliferation was determined by colony formation assay in in FTO knockdown or vector control CRC cells with or without exogenous expression of SLC7A11.

(D) The malondialdehyde (MDA) concentration was detected using MDA assay kits in METTL3 or METTL14 knockdown or vector control CRC cells.

(E) The GSH/GSSG ratio was detected using GSH/GSSG assay kits in METTL3 or METTL14 knockdown or vector control CRC cells.

(F) The malondialdehyde (MDA) concentration was detected using MDA assay kits in METTL3 or YTHDF2 knockdown or vector control CRC cells treated with or without Erastin or RSL3 at indicated day 3.

(G) The GSH/GSSG ratio was detected using GSH/GSSG assay kits in METTL3 or YTHDF2 knockdown or vector control CRC cells treated with or without Erastin or RSL3 at indicated day 3.

(All error bars, mean values ± SEM, p values were determined by unpaired two-tailed Student’s t test of n = 3 independent biological experiments. *p < 0.05; **p < 0.01; ***p < 0.001).

**Figure S6 Identification of Mupirocin as a novel inhibitor of FTO and regulates CRC ferroptosis and tumor growth.**

1. The list of the pipeline to identify top 7 compounds.
2. The total RNA was harvested from HCT116 cells, and then incubated with FTO protein from HCT116 cells by flag pull-down in kinase buffer with or without 2 compounds. The enzymatic activity of FTO was analyzed by dot blotting assay.

(C) Thermal shift analysis the binding of Mupirocin to FTO WT or mutant by western blot analysis.

(D) The structure of Mupirocin and Mupirocin probe.

(E) The total RNA was harvested from HCT116 cells, and then incubated with recombinant FTO protein in kinase buffer with or without Mupirocin probe. The enzymatic activity of FTO was analyzed by dot blotting assay.

(F) The total RNA were harvested from HCT116 cells, and then incubated with recombinant FTO protein in kinase buffer with or without Mupirocin probe. The enzymatic activity of FTO was analyzed by ELISA.

(G) The IC50 of Mupirocin and Mupirocin probe in LoVo were determined by cell number counting assay at indicated day 3.

(H) Volcano plot showing genome-wide mRNA expression in Mupirocin treated or control HCT8 cells.

(I) The gene set enrichment analysis (GSEA) was used to analyze the FTO relative signaling pathways enrichment in Mupirocin treated or control group.

(J) Heatmap indicated the mRNA expression level of ferroptosis-related genes in Mupirocin treated or control HCT8 cells.

(K) The expression of FTO, SLC7A11, and GPX4 were examined by western blotting in Mupirocin treated or control CRC cells at indicated day 3.

(L) The colony formation was determined in CRC cells treated with the indicated doses of Mupirocin.

(M) Cell viability assay detection of the viability of organoids the indicated doses of Mupirocin.

(N) HE, Ki67, FTO, SLC7A11, GPX4, and 4HNE were analyzed in a representative PDX xenograft tumor by IHC (scale bar = 50 μm).

(O) The mice weight was compared between xenograft nude mice with PDX tumor treated with Mupirocin (n=4).

(All error bars, mean values ± SEM, p values were determined by unpaired two-tailed Student’s t test of n = 3 independent biological experiments. *p < 0.05; **p < 0.01; ***p < 0.001).

**Figure S7 Mupirocin enhances the anti-tumor effects of Erastin and RSL3.**

(A) The cell proliferation was determined by cell number counting assay at indicated day 1, day 2, day 3, and day 4 in CRC cells treated with Mupirocin in the absence or presence of Erastin.

(B) The cell proliferation was determined by cell number counting assay at indicated day 1, day 2, day 3, and day 4 in CRC cells treated with Mupirocin in the absence or presence of RSL3.

(C-D) The colony formation was determined in CRC cells treated with Mupirocin in the absence or presence of Erastin.

(E) The cell proliferation was determined by cell number counting assay in FTO knockdown or vector control CRC cells treated with Mupirocin in the absence or presence of Erastin or RSL3 at indicated day 3.

(F) The mice weight was compared between xenograft nude mice with PDX tumor treated with Mupirocin in the absence or presence of Erastin (n=4).

(G) The mice weight was compared between xenograft nude mice with PDX tumor treated with Mupirocin in the absence or presence of RSL3 (n=4).

(All error bars, mean values ± SEM, p values were determined by unpaired two-tailed Student’s t test of n = 3 independent biological experiments. *p < 0.05; **p < 0.01; ***p < 0.001).

**Figure S8 SLC7A11 and GPX4 expression is positively correlated with FTO in patients with colorectal cancer.**

(A-C) Immunohistochemical analysis of FTO, SLC7A11, and GPX4 in a CRC tissues microarray (TMA) containing distant normal tissues (N) and CRC tumor tissues (T) (Cohort: n=44) (scale bar = 50 μm).

(D-F) Quantification of FTO, SLC7A11, and GPX4 expression by Image J software from IHC data.

(G-H) The correlation between SLC7A11, GPX4 expression and FTO expression in CRC tissues (Cohort: n=44).

(I) Proposed model: FTO protects colorectal cancer from ferroptotic cell death through triggering SLC7A11/GPX4 expression.

(All error bars, mean values ± SEM, p values were determined by unpaired two-tailed Student’s t test of n = 3 independent biological experiments. *p < 0.05; **p < 0.01; ***p < 0.001).


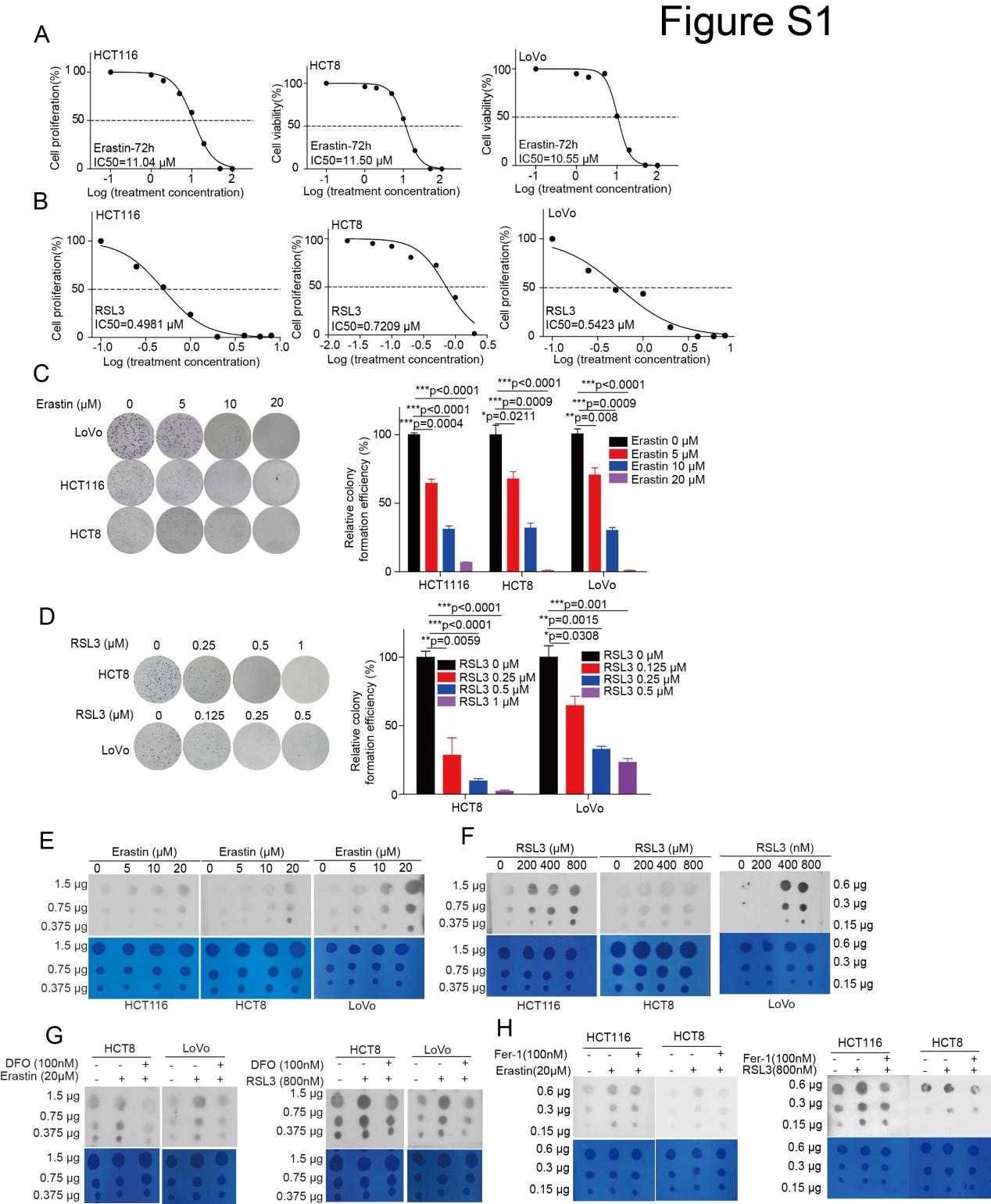


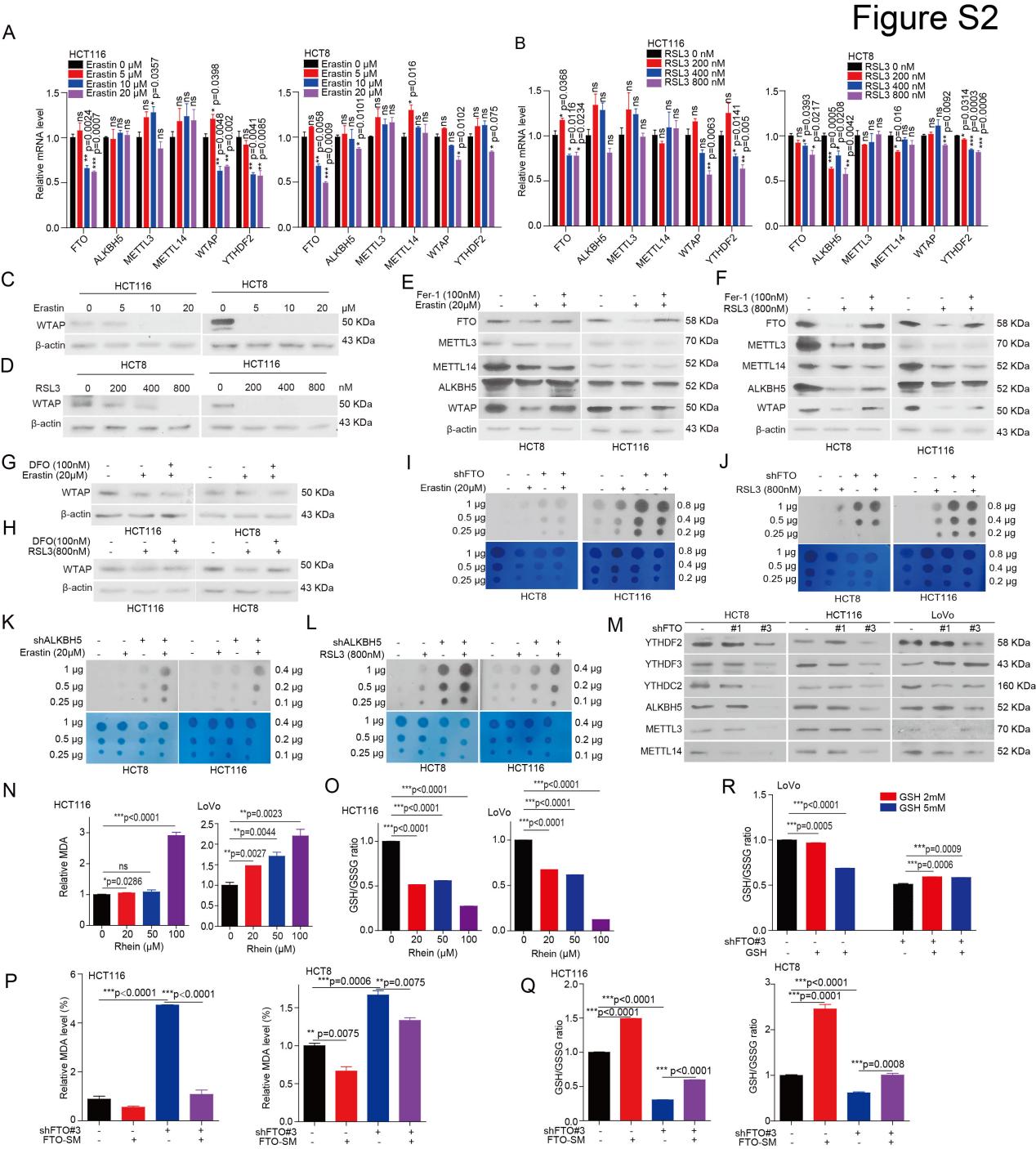


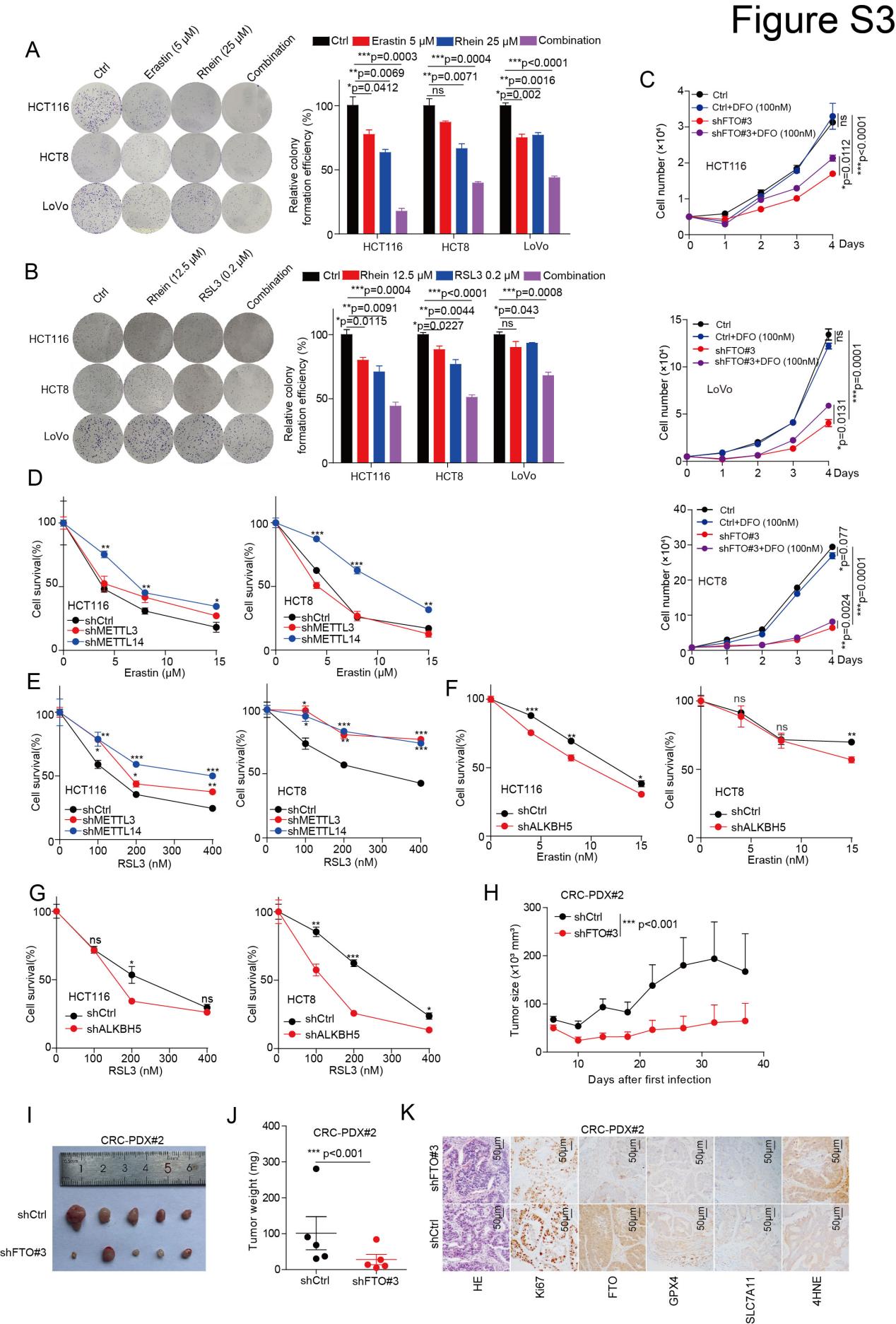


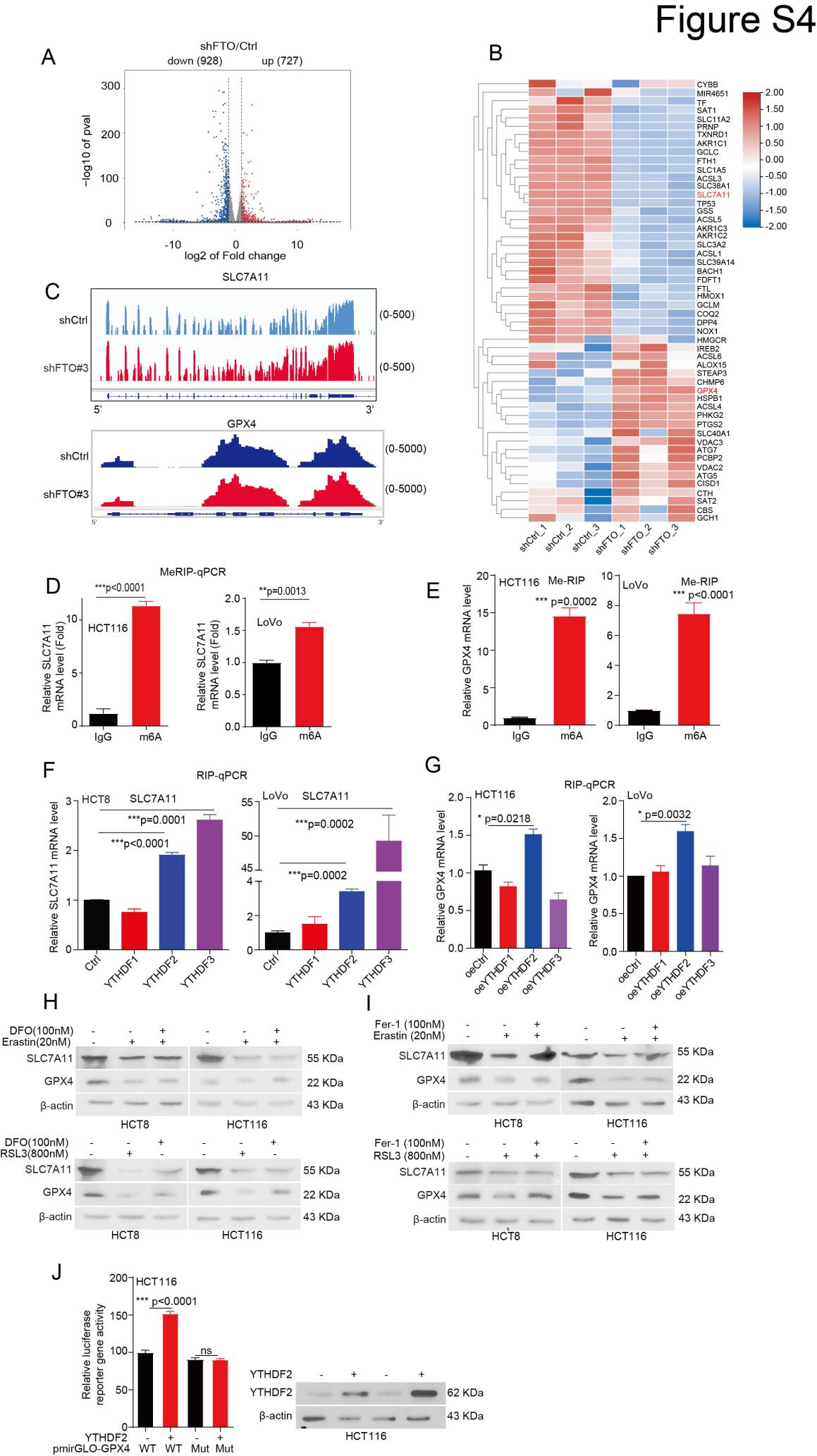


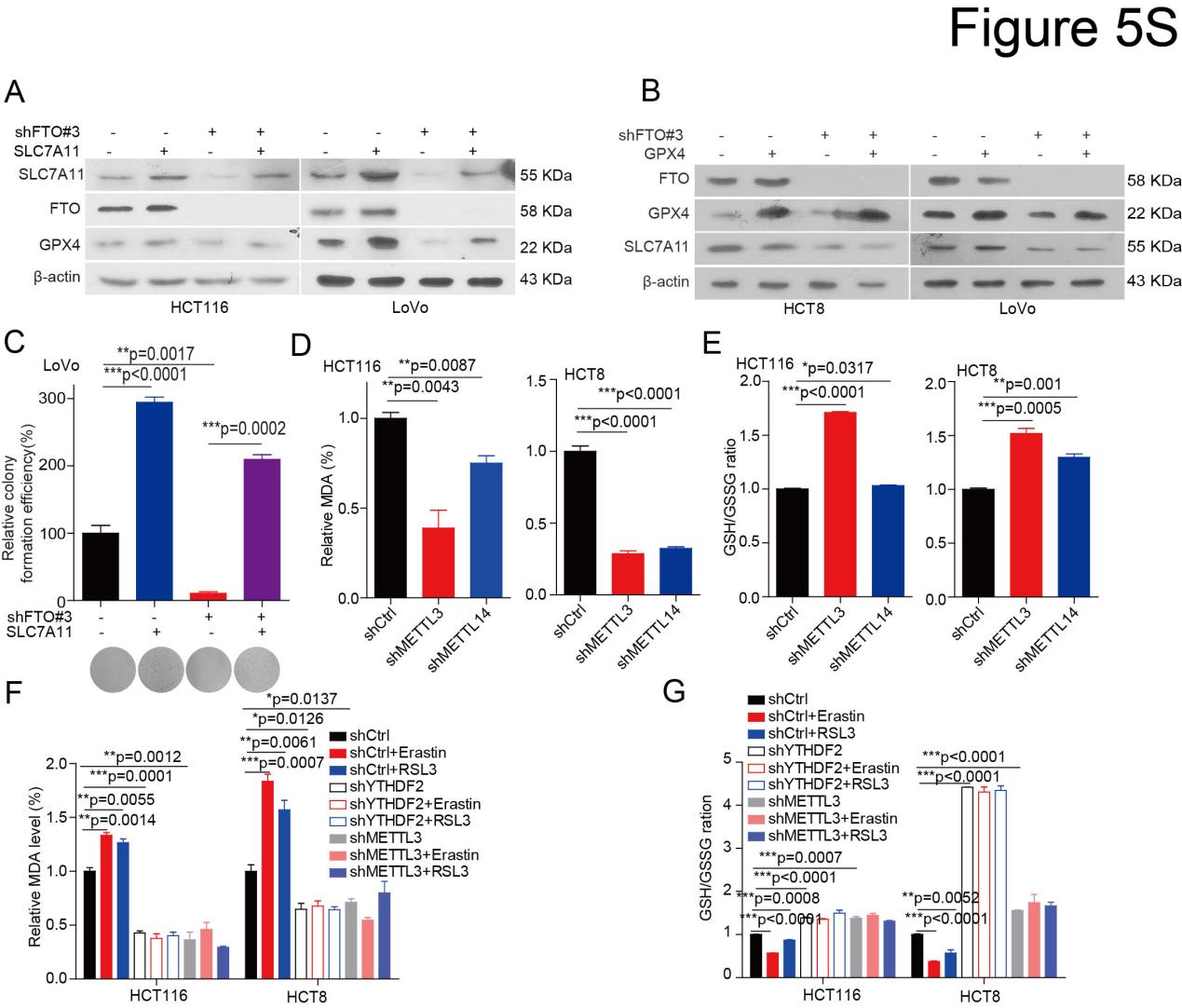


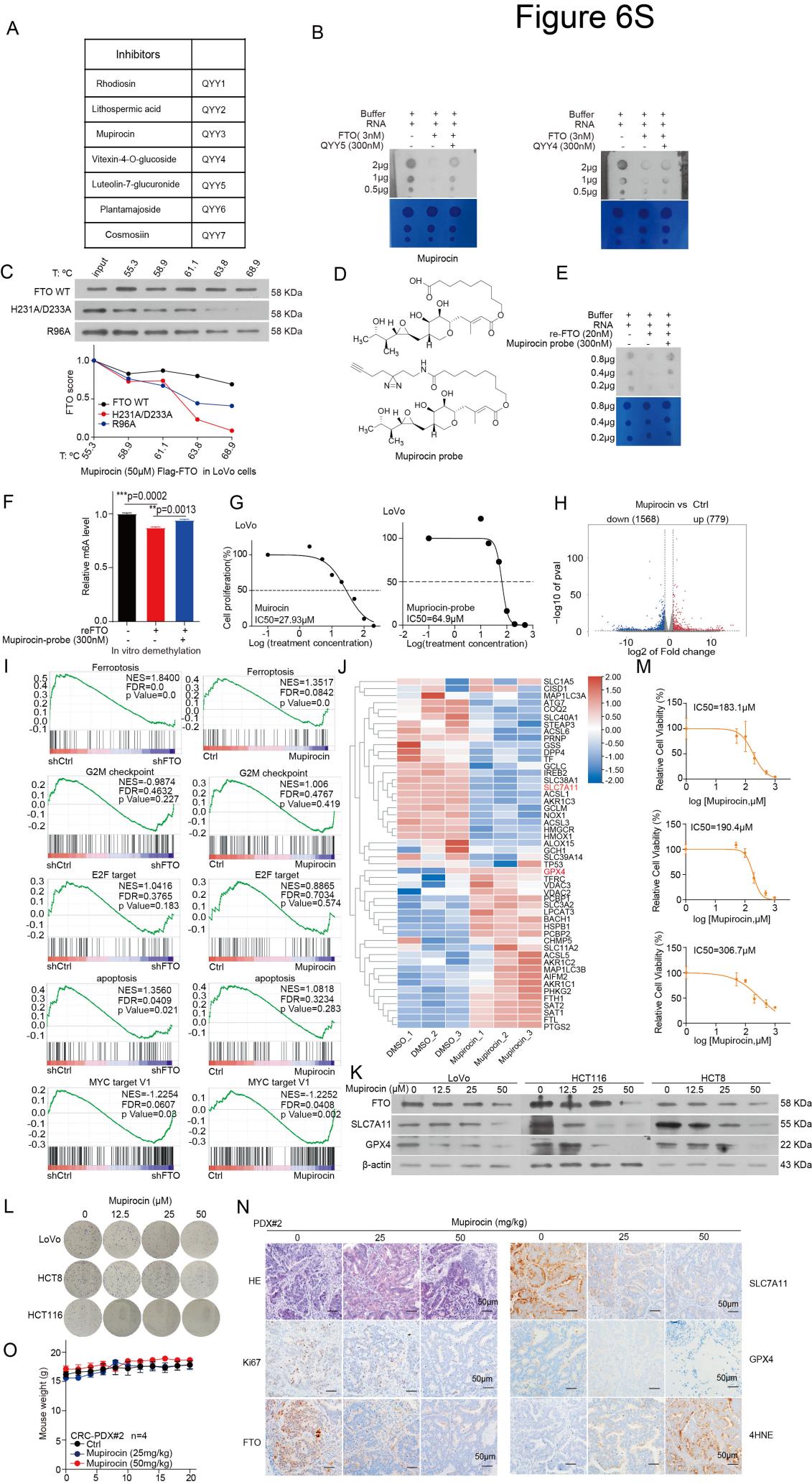


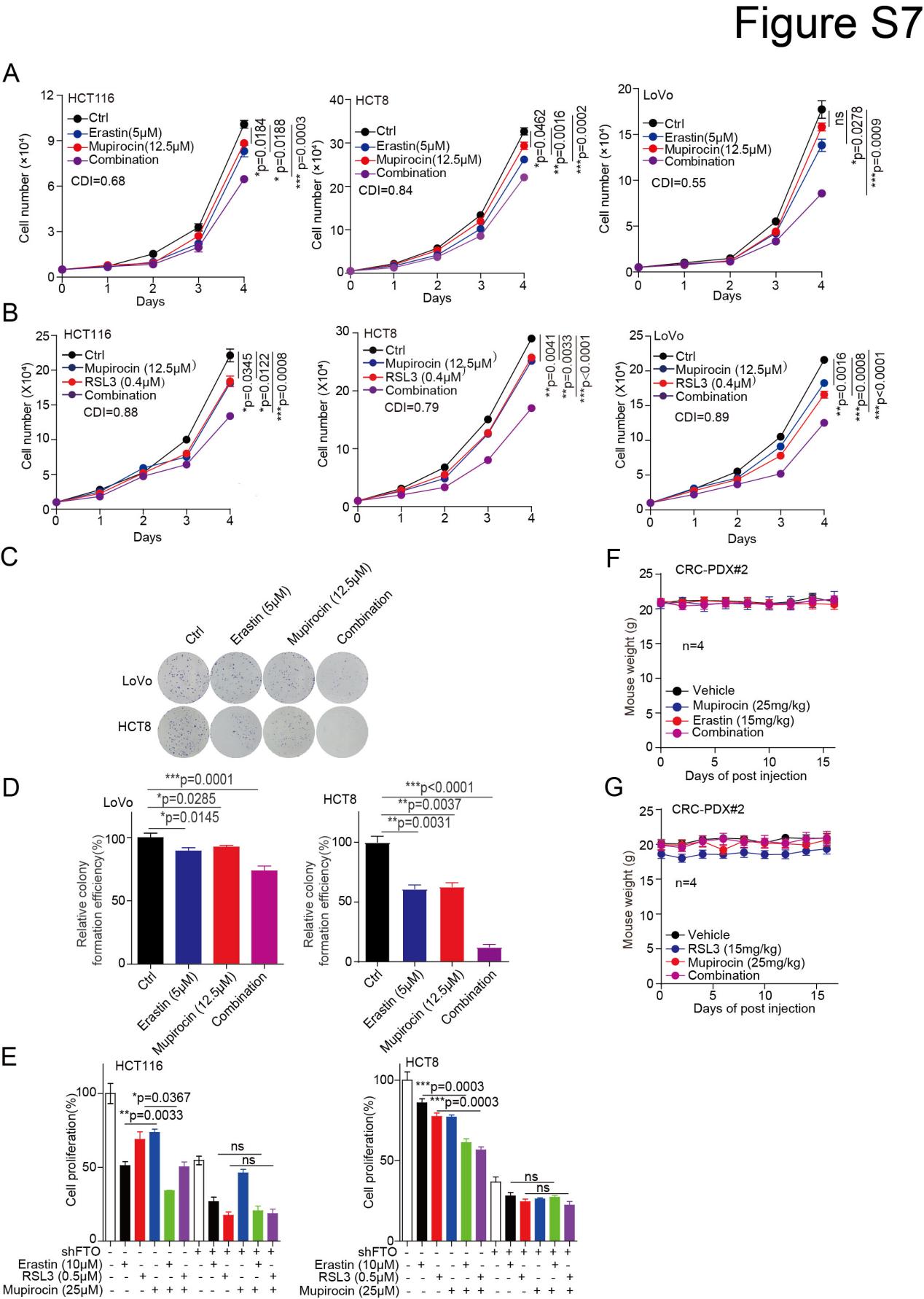


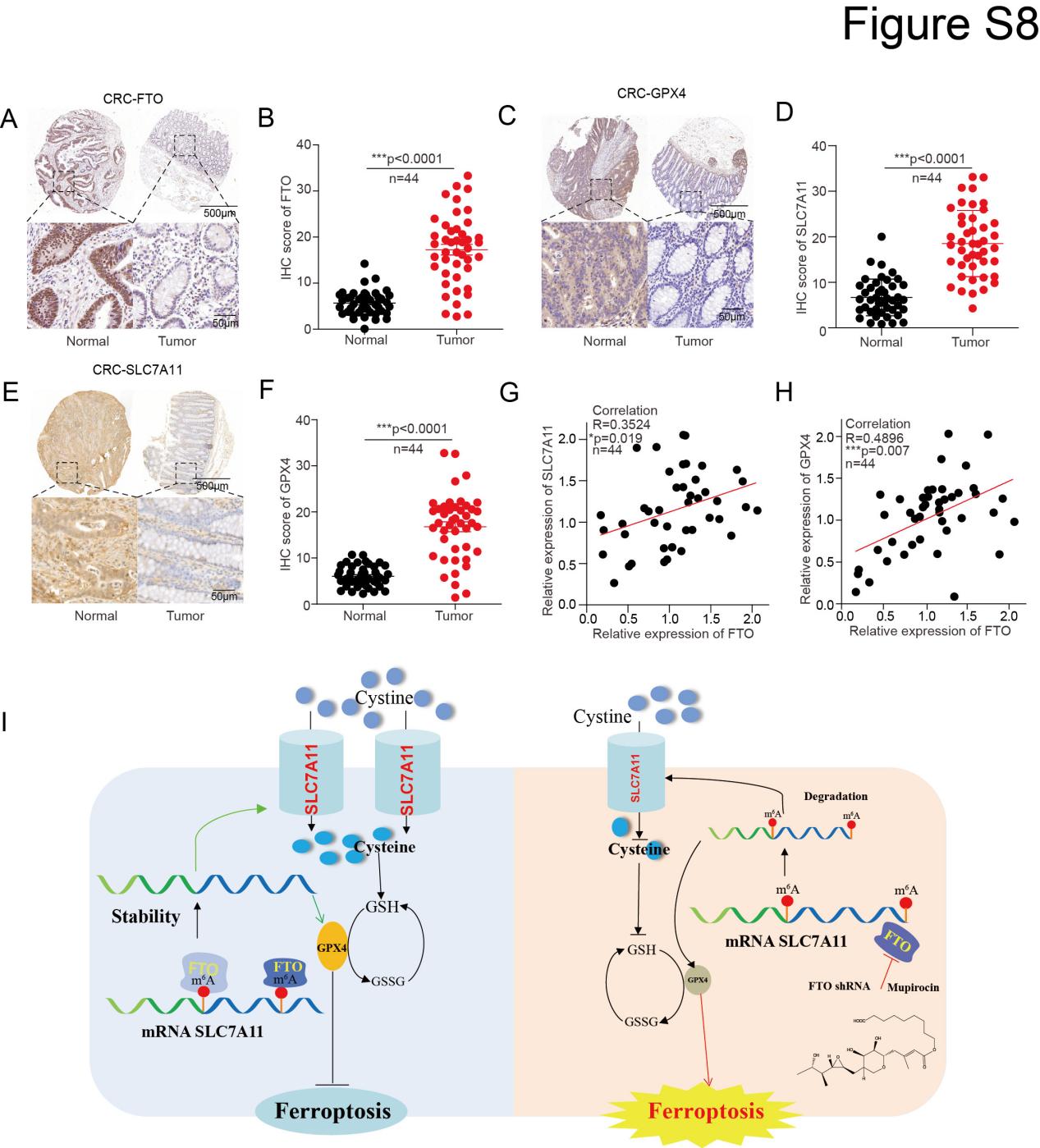

Supplement: Supplementary file 1 — Supplementary Material 1 [file 13046_2024_3032_MOESM1_ESM.docx]
